# Crystal structures of the homologues diethyl and dimethyl (10*H*-indeno­[1,2-*b*]quinoxalin-11-yl)phospho­nate: use of non-spherical scattering factors

**DOI:** 10.1107/S2056989026001842

**Published:** 2026-03-03

**Authors:** Ghada A. Eldeken, Fatma A. El-Samahy, Ehab M. Zayed, Fayez H. Osman, Khaled Mahmoud, Galal H. Elgemeie, Peter G. Jones

**Affiliations:** a Department of Green Chemistry, Chemical Industries Research Institute, National Research Centre, 33 El-Buhouth St., Dokki, Giza, PO 12622, Egypt; b Pharmacognosy Department, National Research Centre, 33 El-Buhouth St., Dokki, Giza, PO 12622, Egypt; c Chemistry Department, Faculty of Science, Capital University, Helwan, Egypt; d Institut für Anorganische und Analytische Chemie, Technische Universität Braunschweig, Hagenring 30, D-38106 Braunschweig, Germany; Vienna University of Technology, Austria

**Keywords:** crystal structure, indeno­quinoxaline, phospho­nate, hydrogen-bonding, non-spherical scattering factors

## Abstract

The two title compounds, **4a** C_19_H_19_N_2_O_3_P and **4b** C_17_H_15_N_2_O_3_P, are not isotypic. The tetra­cyclic ring systems are essentially planar. The phospho­nate groups are similarly oriented. Compound **4a** crystallizes in *P*1 with *Z*′ = 1; mol­ecules are linked in inversion-symmetric pairs by classical hydrogen bonds N—H⋯O=P, forming rings of graph set *R*^2^_2_(12). Compound **4b** crystallizes in *P*2_1_/*c* with *Z*′ = 2; the two independent mol­ecules are linked by a hydrogen-bond system analogous to that of **4a**, but the ring systems subtend an inter­planar angle of 55.84 (1)°. For **4a**, the hydrogen bonding combines with ring stacking in pairs to form a ribbon structure parallel to the *b* axis. For **4b**, the classical hydrogen bonding combines with two ‘weak’ hydrogen bonds C—H⋯O to form a layer structure parallel to the *ac* plane. Non-spherical atom scattering factors were employed (using the program *NoSpherA2*) to avoid the problem of badly fitting reflections in high-resolution data (2θ_max_*ca* 105° using Mo *K*α radiation).

## Abstract 2

The tetra­cyclic ring systems of the two title structures are essentially planar. Mol­ecules are linked by hydrogen bonds N—H⋯O=P, forming rings of graph set *R*^2^_2_(12).

## 1. Chemical context

Nitro­gen-containing heterocycles such as the indeno­[1,2-*b*]quinoxaline moiety and related derivatives have attracted considerable attention in synthetic and medicinal chemistry. Many possess biological activity and are of therapeutic value, involving properties such as anti-inflammatory (Schepetkin *et al.*, 2019 ▸), anti­microbial (Sawant *et al.*, 2025 ▸), acetyl­cholinesterase (AChE) inhibitory activity (Akondi *et al.*, 2017 ▸), anti­tumor activity (Tseng *et al.*, 2016 ▸; Saravana Mani *et al.*, 2018 ▸), α-glucosidase inhibition (Khan *et al.*, 2014 ▸), or c-Jun N-terminal kinase (JNK) inhibition (Schepetkin *et al.*, 2012 ▸, 2019 ▸). They can also be used as acid corrosion inhibitors for mild steel surfaces (Obot & Obi-Egbedi, 2010 ▸).

Phospho­rus is the one of the most essential elements of life and is widely distributed in nature. Phospho­rus-containing drugs constitute an important class of therapeutic agents targeting a wide range of diseases (Karl, 2000 ▸; Yu *et al.*, 2020 ▸; Engel, 1992 ▸). Organo­phospho­rus compounds have numerous applications in agriculture (Okoroiwu & Iwara, 2018 ▸; Lu *et al.*, 2023 ▸), veterinary science (Marrs, 2003 ▸) and medicine.

**Figure 1:**
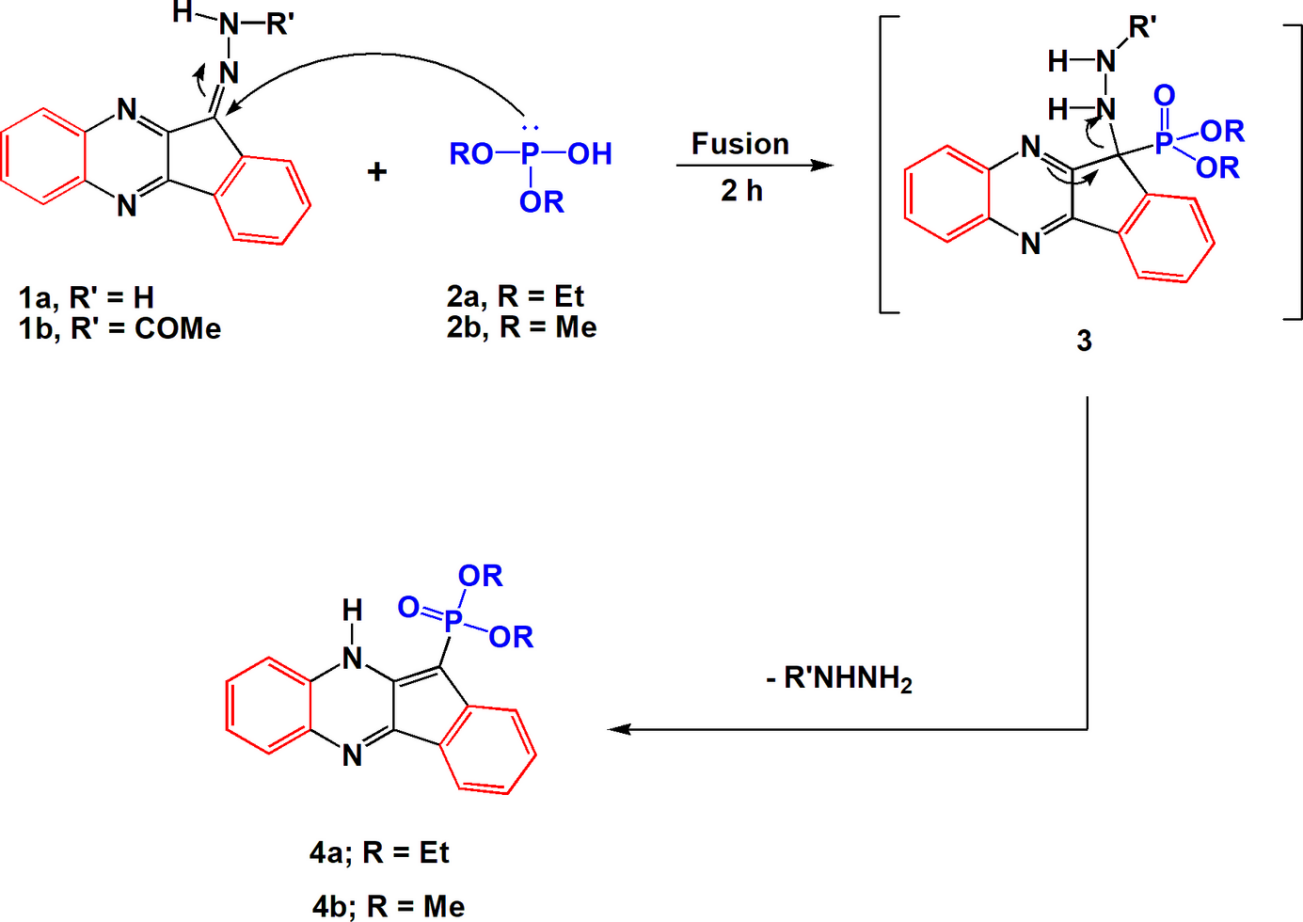
The synthesis scheme of compounds **4a** and **4 b**.

In a continuation of our work on compounds with the indeno­[1,2-*b*]quinoxaline moiety (Eldeken *et al.*, 2022 ▸; El-Samahy *et al.*, 2023 ▸), we have focused on synthesizing new phospho­nates as potentially active compounds, and studying their biological activities, in particular as anti­cancer agents. The aim of the current study was to produce new indeno[1,2-*b*]quinoxaline hybrids. The reaction of 11-hydrazineyl­idene-11*H*-indeno­[1,2-*b*]quinoxaline **1a** or *N*′-(11*H*-indeno­[1,2-*b*]quinoxalin-11-yl­idene)acetohydrazide **1b** with dialkyl phosphites **2a,b** without solvent led to the synthesis of the unexpected products diethyl (10*H*-indeno­[1,2-*b*]quinoxalin-11-yl)phospho­nate **4a** and dimethyl (10*H*-indeno­[1,2-*b*]quinoxalin-11-yl)phospho­nate **4b** in good yield. The proposed mechanism for the formation of **4** (Fig. 1 ▸) shows the nucleophilic attack by the phosphite phospho­rus atom on the azine C=N bond attached to the indeno­quinoxaline moiety in **1** to form the inter­mediates **3**, which then undergo the elimination of hydrazine derivatives with formation of the phospho­nates **4.** The structures of **4** were inferred from the spectroscopic data, but, in order to establish the structure of the products unambiguously, their crystal structures were determined and are reported here.
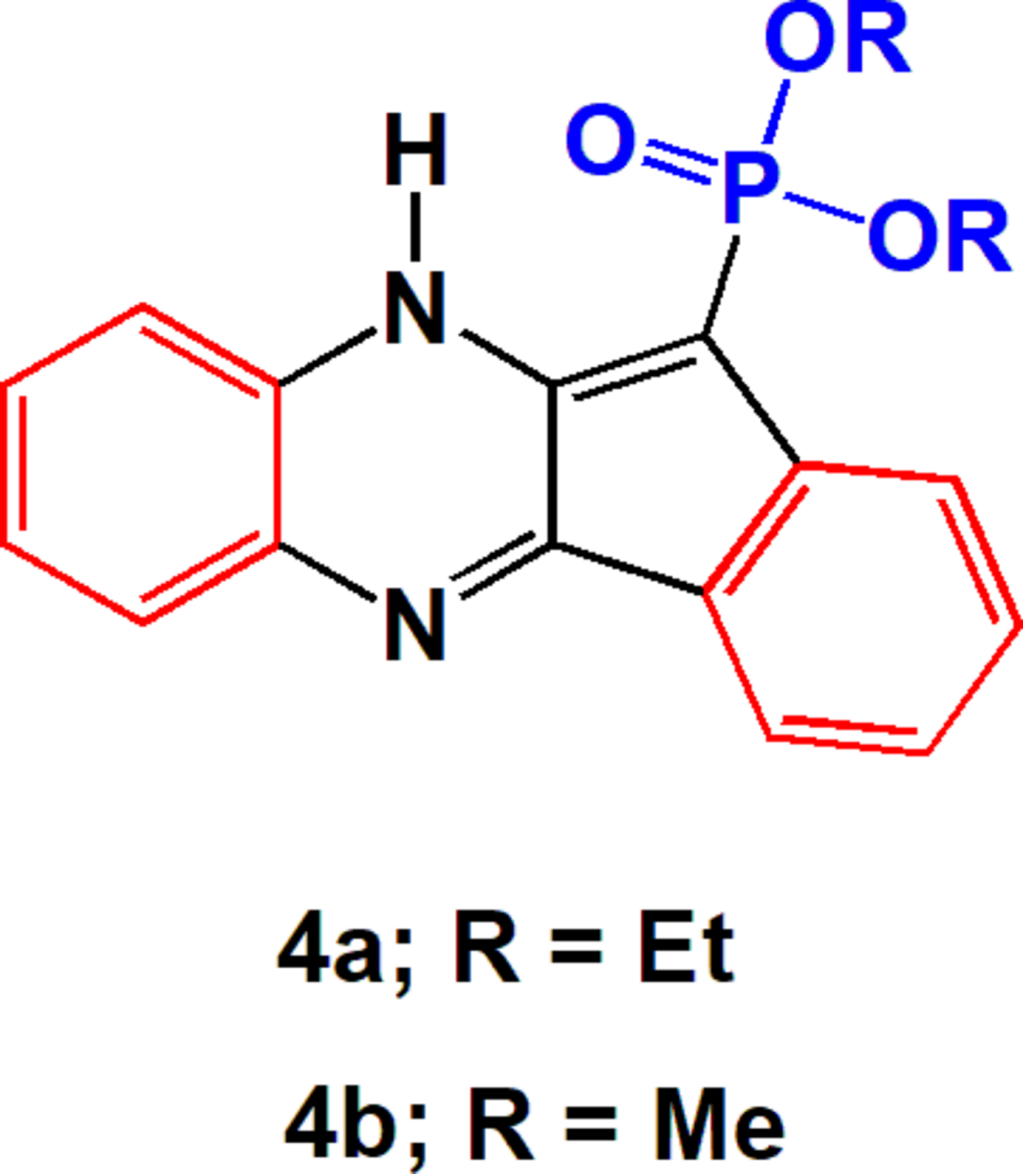


## 2. Structural commentary

**Figure 2:**
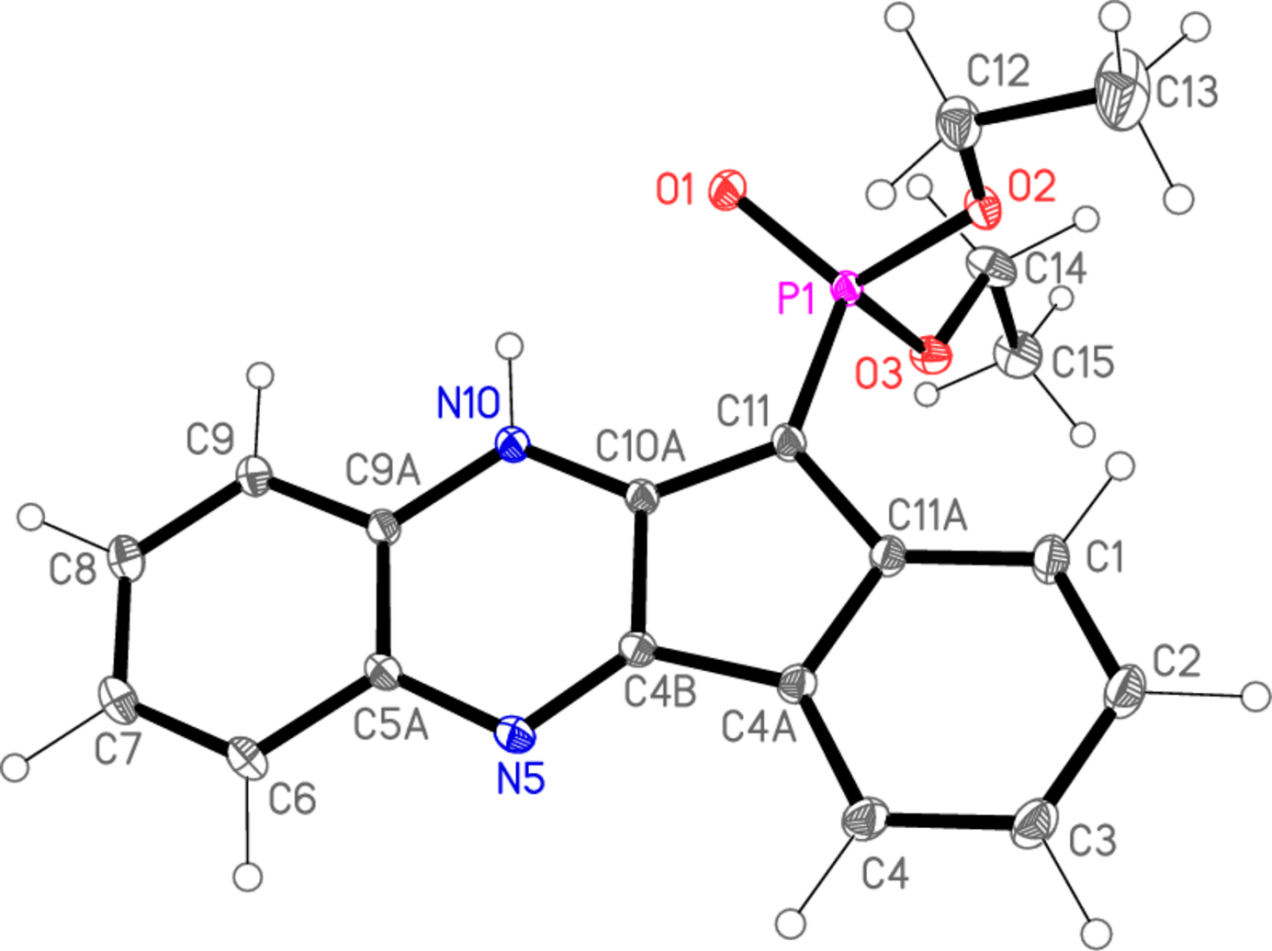
The mol­ecule of compound **4a** in the crystal. Ellipsoids correspond to 50% probability levels.

Compounds **4a** and **4b** are not isotypic. Their mol­ecular structures are shown in Figs. 2 ▸ and 3 ▸ respectively. Selected mol­ecular dimensions are given in Tables 1 ▸ and 2 ▸ respectively. Compound **4a** crystallizes with one mol­ecule in the asymmetric unit while compound **4b** crystallizes with two mol­ecules in the asymmetric unit. In both structures, pairs of mol­ecules are connected by two N—H⋯O=P hydrogen bonds (Tables 3 ▸ and 4 ▸) to form rings of graph set 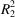
(12); for more details see Section 3. Atoms of the second mol­ecule of **4b** are denoted by primes (’) where possible (except for C10*B* and C11*B*).

**Figure 4:**
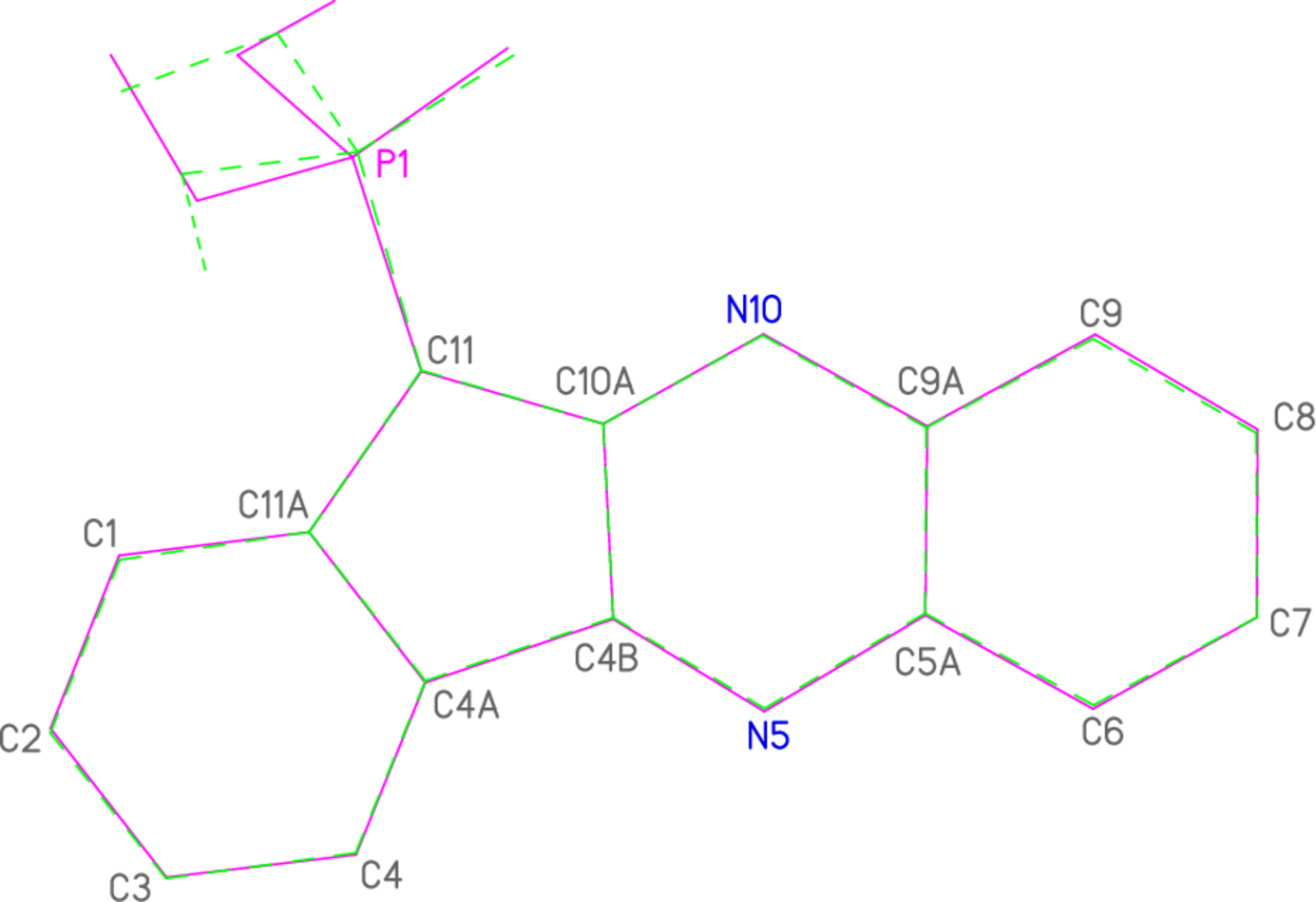
A least-squares fit of the two mol­ecules of **4b**. Fitted atoms are labelled; their r.m.s. deviation is 0.07 Å.

The presence of the five-membered ring necessarily introduces some distortions to the system, in particular the large exocyclic angles at C4*A*, C4*B*, C10*A*, C11*A* and C11. The 17-atom ring systems are essentially planar, with r.m.s. deviations (Å) of 0.015 for **4a**, 0.017 and 0.062 for **4b**. The inter­planar angle in **4b** is 55.84 (1)°. The dimensions of the phospho­nate groups are broadly as expected, with formal P=O1 double bonds some 0.1 Å shorter than the P—O single bonds and most formal O=P—O and O=P—C angles appreciably wider than their O—P—O and O—P—C counterparts (but with exceptions for O—P—C of the second mol­ecule of **4b**, for reasons that are not clear). The phospho­nate groups, except for the methyl groups of the second mol­ecule of **4b**, are similarly oriented in the three mol­ecules (one of **4a** and two of **4b**), as can be seen from the torsion angles in Tables 1 ▸ and 2 ▸, although the signs of these angles are (by chance) reversed in the two structures. A better fit (no torsion angle differences larger than *ca*. 11°) of the two mol­ecules of **4b** is obtained using the coordinates directly rather than inverting one of the mol­ecules, so that the mol­ecules are better described as rotated rather than inverted to each other (in contrast to **4a**, see Section 3). This fit is shown in Fig. 4 ▸.

## 3. Supra­molecular features

**Figure 5:**
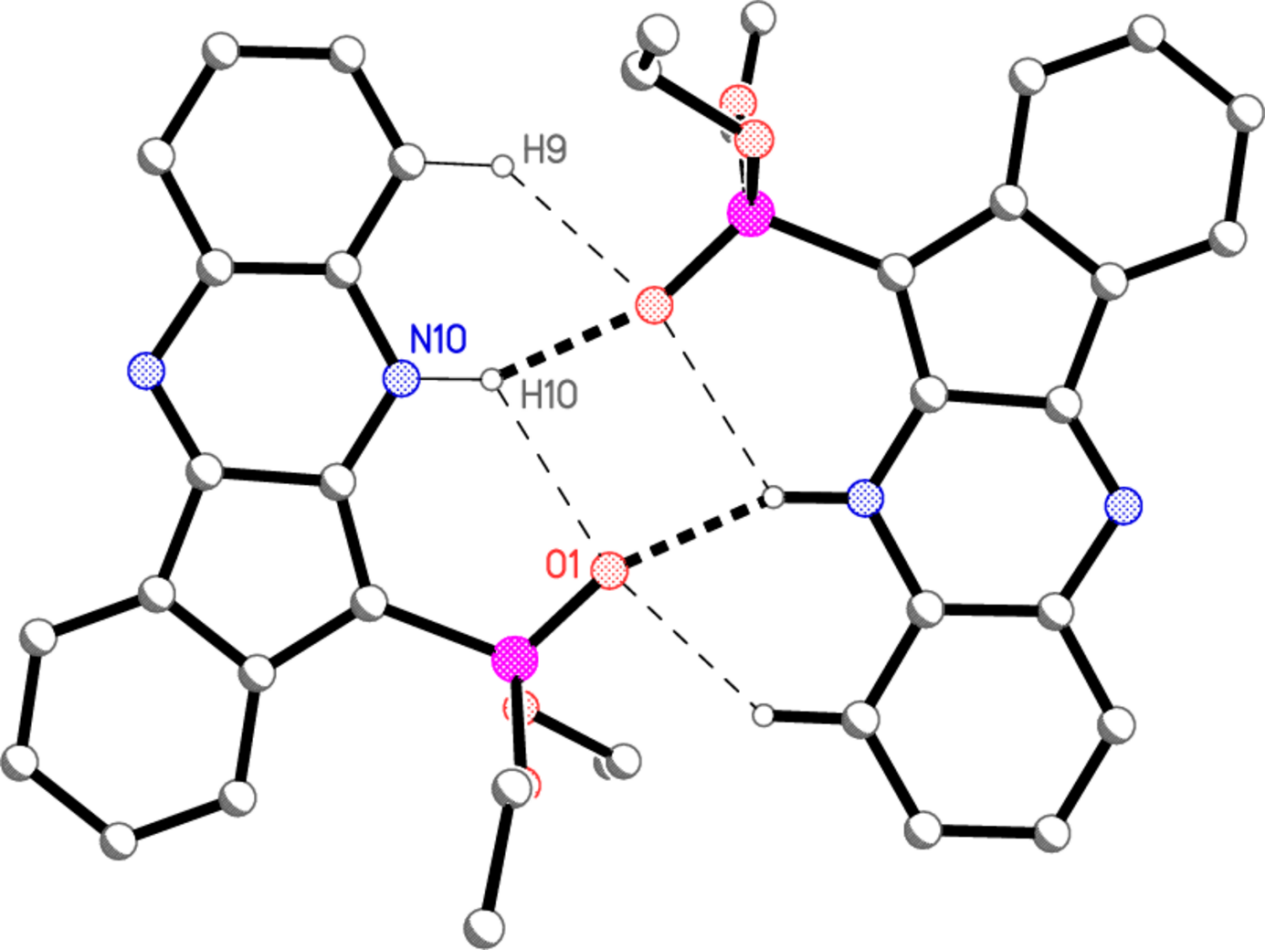
The dimeric unit of compound **4a**, showing the classical hydrogen bonds (thick dashed lines), with a weaker component of a three-centre system and a weak C—H⋯O contact (thin dashed lines). Hydrogen atoms not involved in H bonding are omitted. Atom labels indicate the asymmetric unit. See Section 3 for more information.

Hydrogen bonds are listed in Tables 3 ▸ and 4 ▸. It should be noted that, when using *NoSpherA2* (see Section 6), the C—H and N—H bond lengths do not show the usual apparent shortening associated with X-ray measurements, so that the hydrogen-bond lengths from the hydrogen donors to the acceptor atoms are appreciably shorter than conventional values. The mol­ecules of **4a** are connected by the same type of hydrogen bonds as for **4b** (see above), with the same graph set, but via an inversion operator, so that the ring systems of the dimeric unit are necessarily parallel (in contrast to **4b**). For both compounds, two additional types of hydrogen bond are observed: intra­molecular N10—H10⋯O=P, which may be seen as a weaker component of a three-centre hydrogen bond, and C9—H9⋯O=P, ‘weak’ hydrogen bonds that presumably provide additional consolidation. Neither type is drawn explicitly in Fig. 2 ▸ or 3, but the additional contacts are shown for the dimer of **4a** (Fig. 5 ▸).

**Figure 6:**
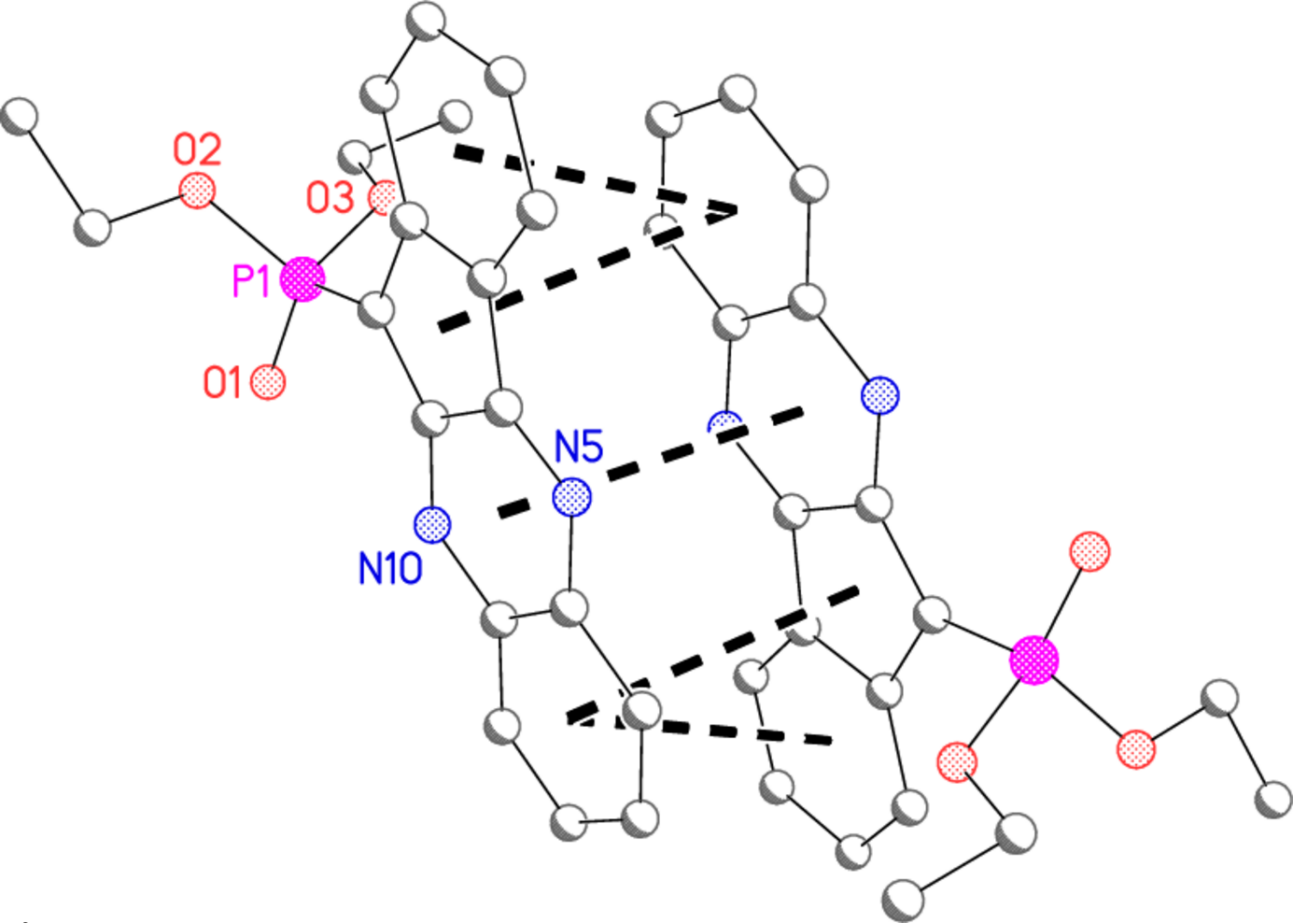
A loosely ‘stacked’ dimer of **4a**, with inter­centroid contacts shown as thick dashed lines. Hydrogen atoms are omitted. N.B. This is a different dimer from that shown in Fig. 5 ▸ (*cf.* operators in text).

**Figure 7:**
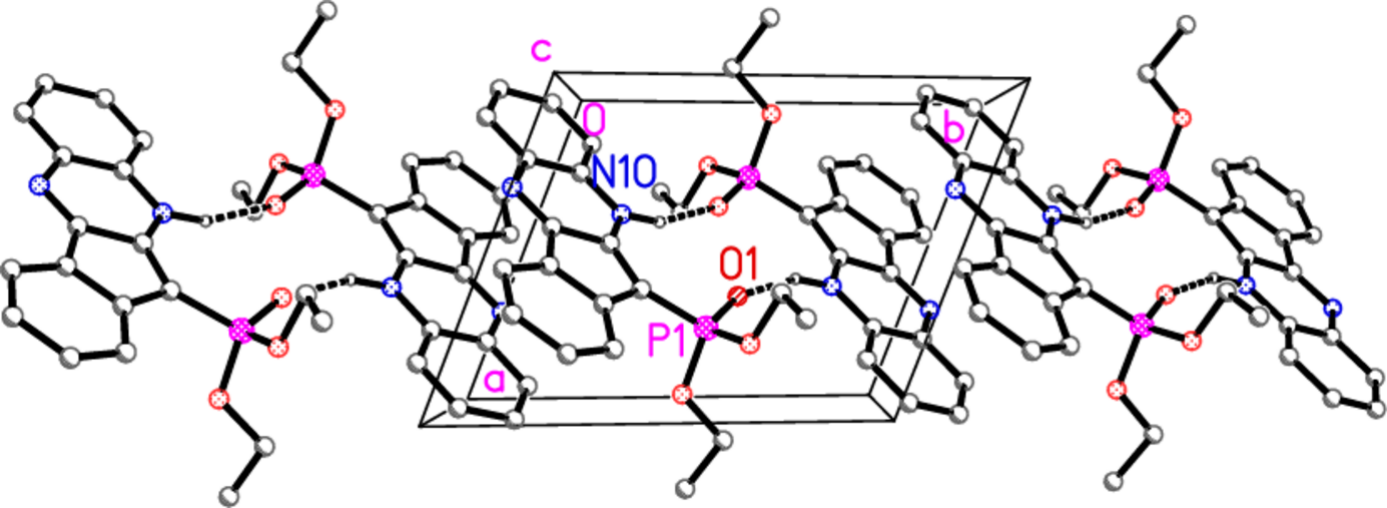
Packing of compound **4a** viewed parallel to the *c* axis, showing chains of hydrogen-bonded dimers parallel to the *b* axis. Hydrogen atoms not involved in hydrogen bonding are omitted. Stacking contacts are not drawn explicitly. Labels correspond to atoms in the asymmetric unit.

The packing of **4a** is otherwise somewhat lacking in major features, and the choice of inter­actions for packing diagrams is necessarily subjective. The ring mol­ecules associate weakly via the inversion operator 1 − *x*, −*y*, 1 − *z* to form stacked pairs (Fig. 6 ▸), but the distances between centroids (*Cg*) are quite long; denoting the rings of Fig. 2 ▸ from right to left as *A*–*D*, the contacts are *CgA*⋯*CgD* = 3.7636 (2), *CgB*⋯*CgD* = 3.6443 (2) and *CgC*⋯*CgC* = 3.6652 (2) Å, with slippages (offsets) of 1.46, 1.15 and 1.36 Å, respectively. The hydrogen bonding and stacking combine to form chains of mol­ecules parallel to the *b* axis (Fig. 7 ▸). There is also stacking of rings *C* and *D* via the operator −*x*, −*y*, 1 − *z*, with *CgC*⋯*CgD* = 3.6902 (2) and *CgD*⋯*CgD* = 3.7409 (2) Å and slippages of 1.36 and 1.49 Å, respectively. Finally, pairs of mol­ecules are connected via the short H⋯π contact C13—H13*B*⋯*CgA*(1 − *x*, 1 − *y*, 2 − *z*), with H⋯π = 2.60 Å and C—H⋯π = 177°.

**Figure 8:**
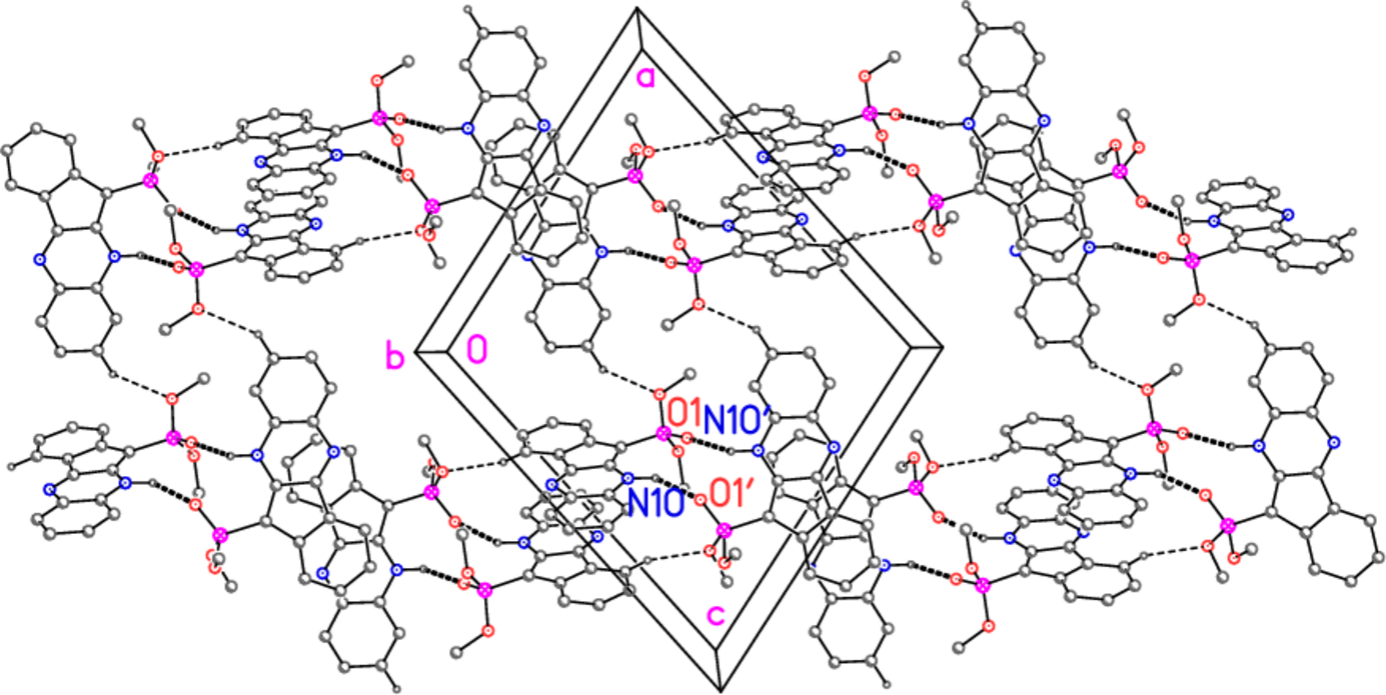
Packing of compound **4b**: the layer structure viewed parallel to the *b* axis. Classical hydrogen bonds are indicated by thick dashed lines and ‘weak’ C—H⋯O hydrogen bonds by thin dashed lines. Labels correspond to atoms in the asymmetric unit.

Compound **4b** also displays a somewhat featureless packing except for its classical hydrogen bonds. There is no face-to-face stacking of the ring systems except for the isolated contacts *CgA*⋯*CgD*(−*x*, 1 − *y*, 1 − *z*) = 3.3062 (1) and *CgA*’⋯*CgD*’(1 − *x*, 1 − *y*, 1 − *z*) = 3.5278 (1) Å, with offsets 0.73 and 0.81 Å, respectively, unless *Cg*⋯*Cg* contacts of up to *ca*. 2.9 Å are accepted (*cf*. Fig. 8 ▸, where the more loosely stacked pairs of ring systems can be recognized; we note that the analysis of possible stacking inter­actions is hampered by the fact that inter­centroid distances are exactly defined, whereas the perhaps more important perpendicular distances between the ring systems may be shorter, but are not exactly defined, especially if the ring systems are not exactly parallel by symmetry). There are also three ‘weak’ hydrogen bonds of the form C—H⋯O or C—H⋯N (Table 4 ▸). The C—H⋯O contacts combine with the classical hydrogen bonds to form a layer structure parallel to the *bc* plane (Fig. 8 ▸), with mol­ecules linked by H4⋯O3′ parallel to [101] (horizontal in Fig. 8 ▸) and by H8′⋯O2 parallel to [10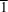] (vertical in Fig. 8 ▸).

## 4. Database survey

Searches were conducted using CSD Version 6.00 (Groom *et al.*, 2016 ▸) and the ConQuest routine (Bruno *et al.*, 2002 ▸), Version 2025.1.1, and showed that the structures of **4a** and **4b** may be regarded as novel. A search for the same tetra­cyclic ring system as in **4a** and **4b**, with the coordination numbers of all carbon atoms set to 3, but no restrictions on those of the nitro­gen atoms, gave no hits with an NH group at N10 (using the atom numbering of **4a** and **4b**). Removing the requirement for a hydrogen atom at N10 gave 17 hits, all with no hydrogen atom at N10 but a double-bonded substituent at C11 (*e.g*. 7,8-dimethyl-11*H*-indeno­[1,2-*b*]quinoxalin-11-one, refcode OJIRUX; Chen *et al.*, 2021 ▸) rather than the singly-bonded phospho­nate group of **4a** and **4b**.

## 5. Synthesis and crystallization

A mixture of **1** (0.01 mol) and dialkyl phosphites **2** (3 ml) was heated for 2 h at 353 K (Fig. 1 ▸). After completion of the reaction (TLC), excess volatile material was removed under vacuum and the resulting residue was purified chromatographically on silica gel.

***Diethyl (10H-indeno­[1,2-b]quinoxalin-11-yl)phospho­n­ate (4a):*** Elution with *n*-hexa­ne/ethyl acetate (60/40, *v*/*v*) afforded pure phospho­nate **4a**, which was recrystallized from ethyl acetate as very dark red–brown or purple, effectively black, crystals with a ridge-tile habit. Clearly these were twinned, but single crystals were cut from the twins without great difficulty, whereby only one side of the ‘V’ cross-section was used. Dark red–brown solid; yield 65%; m.p. 418 K; IR (KBr, cm^−1^): *ν* 3140 (NH), 2919 (aromatic and aliphatic CH), 1600 (C=O, C=N), 1192 (P=O), 1015 (P—O—C) cm^−1^; ^1^H NMR (500 MHz, DMSO-*d*_6_): *δ* 1.19, 1.21 [2 *t*, *J_HH_* = 7.0 Hz, P(OCH_2_CH_3_)_2_], 3.95, 3.96 [2 *q*, *J_HH_* = 7.0 Hz, P(OCH_2_CH_3_)_2_], 7.56–8.21 (*m*, 8 ArH), 12.00 (*s*, NH) ppm; ^13^C NMR (125 MHz, DMSO-*d*_6_): *δ* 16.8 (P(OCH_2_CH_3_)_2_, 62.9, 63.6 [P(OCH_2_CH_3_)_2_], 100.0, 118.2, 119.3, 122.6, 129.2, 129.3, 129.4, 129.7, 129.9, 130.0, 130.6, 132.3, 145.3, 157.6 and 162.6 ppm; EI MS *m*/*z* (%) 354 (*M*^+^, 100%); Analysis calculated for C_19_H_19_N_2_O_3_P (354.35): C 64.40, H 5.40, N 7.91, P 8.74; found: C 64.49, H 5.51, N 7.80, P 8.86%.

***Dimethyl (10H-indeno­[1,2-b]quinoxalin-11-yl)phospho­n­ate (4b):*** Elution with *n*-hexa­ne/ethyl acetate (50/50, *v*/*v*) afforded pure phospho­nate **4b**, which was recrystallized from ethyl acetate as very dark red–brown or purple, effectively black, inter­grown clumps, from one of which a single-crystalline fragment was separated using a razor blade. Dark red–brown solid; yield 65%; m.p. 453 K; IR (KBr, cm^−1^): ν 3139 (NH), 3007 (aromatic CH), 2918 (aliphatic CH), 1601 (C=N), 1216 (P=O), 1022 (P—O—C) cm^−1^; ^1^H NMR (500 MHz, CDCl_3_): *δ* 3.76, 3.78 [2 *d*, *^3^J_PH_* = 11.2 Hz, 6 H, P(OCH_3_)_2_], 7.52–8.38 (*m*, 8 ArH), 11.17 (*s*, NH) ppm; ^13^C NMR (125 MHz, CDCl_3_): δ 52.5, 53.5 [P(OCH_3_)_2_], 116.4, 119.0, 121.9, 122.9, 123.3, 123.7, 127.0, 127.9, 129.0, 129.3, 129.7, 130.2, 130.3, 131.7, 132.1, 139.8 and 155.7 ppm; EI MS *m*/*z* (%) 326 (*M*^+^,7%); Analysis calculated for C_17_H_15_N_2_O_3_P (326.29): C 62.58, H 4.63, N 8.59, P 9.49; found C 62.69, H 4.77, N 8.48, P 9.61%.

## 6. Refinement

Details of data collection and structure refinement for **4a** and **4b** are summarized in Table 5 ▸. The crystals diffracted strongly, and data were accordingly collected to 2*θ*_max_ of *ca*. 105°. Both structures were solved using *SHELXT* (Sheldrick, 2015*a* ▸). Normal refinement with *SHELXL2019/3* (Sheldrick, 2015*b* ▸) led to *wR*2 values of 0.1098 and 0.1178 respectively, with *R*1 0.0346 and 0.0391 respectively. Although these values are entirely satisfactory, there was a problem with badly-fitting reflections (listed by *SHELXL* as ‘Most Disagreeable Reflections’). Thus for **4a** there were 28 reflections with Δ/σ values of 7–12.6, whereas for **4b** there were 23 reflections with Δ/σ values of 7–11. All the bad reflections were weak but significant, and had *F*_o_^2^ >> *F*_c_^2^. In a recent paper (Jones, 2025 ▸) one of us has commented that this seems to be a general effect for strongly scattering organic structures measured to high diffraction angles, and is probably attributable to the use of spherical scattering factors. Accordingly, the program *NoSpherA2* (Kleemiss *et al.*, 2021 ▸, and references therein) was used for the refinement; it runs under the *Olex2* platform (Dolomanov *et al.*, 2009 ▸; Bourhis *et al.*, 2015 ▸). We summarize its mode of operation (involving the calculation of non-spherical scattering factors for each atom) in our previous paper (Jones, 2025 ▸), but the original publications should be consulted for full details. The *wR*2 and *R*1 values were, necessarily, greatly reduced compared to the standard refinement; more importantly, the number and severity of ‘bad’ reflections were reduced drastically, so that we prefer these refinement models to the conventional refinements (for **4a**, only three reflections had Δ/σ > 5, and for **4b** the worst reflection had Δ/σ 4.2). However, the extremely low su’s of mol­ecular dimensions (Tables 1 ▸ and 2 ▸) should probably be inter­preted cautiously. It should be noted that the default for *Olex2*/*NoSpherA2* refinement is for hydrogen atoms to be refined anisotropically; this was also the case for **4a** and **4b**, and seems to have given sensible results. However, the ellipsoids for some hydrogen atoms, especially of the ethyl groups in **4a**, are then quite large, so that we draw these atoms as spheres of arbitrary radius in Figs. 2 ▸ and 3 ▸ for the sake of clarity. We note in passing that the particularly large numbers of ‘bad’ reflections observed here and by Jones (2025 ▸) for conventional refinement all involve structures of organic compounds with slightly heavier atoms such as sulfur or phospho­rus. It remains to be seen if this observation can be generalized.

## Supplementary Material

Crystal structure: contains datablock(s) 4a, 4b, global. DOI: 10.1107/S2056989026001842/wm5789sup1.cif

Structure factors: contains datablock(s) 4a. DOI: 10.1107/S2056989026001842/wm57894asup2.hkl

Structure factors: contains datablock(s) 4b. DOI: 10.1107/S2056989026001842/wm57894bsup3.hkl

CCDC references: 2532769, 2532770

Additional supporting information:  crystallographic information; 3D view; checkCIF report

## Figures and Tables

**Figure 3:**
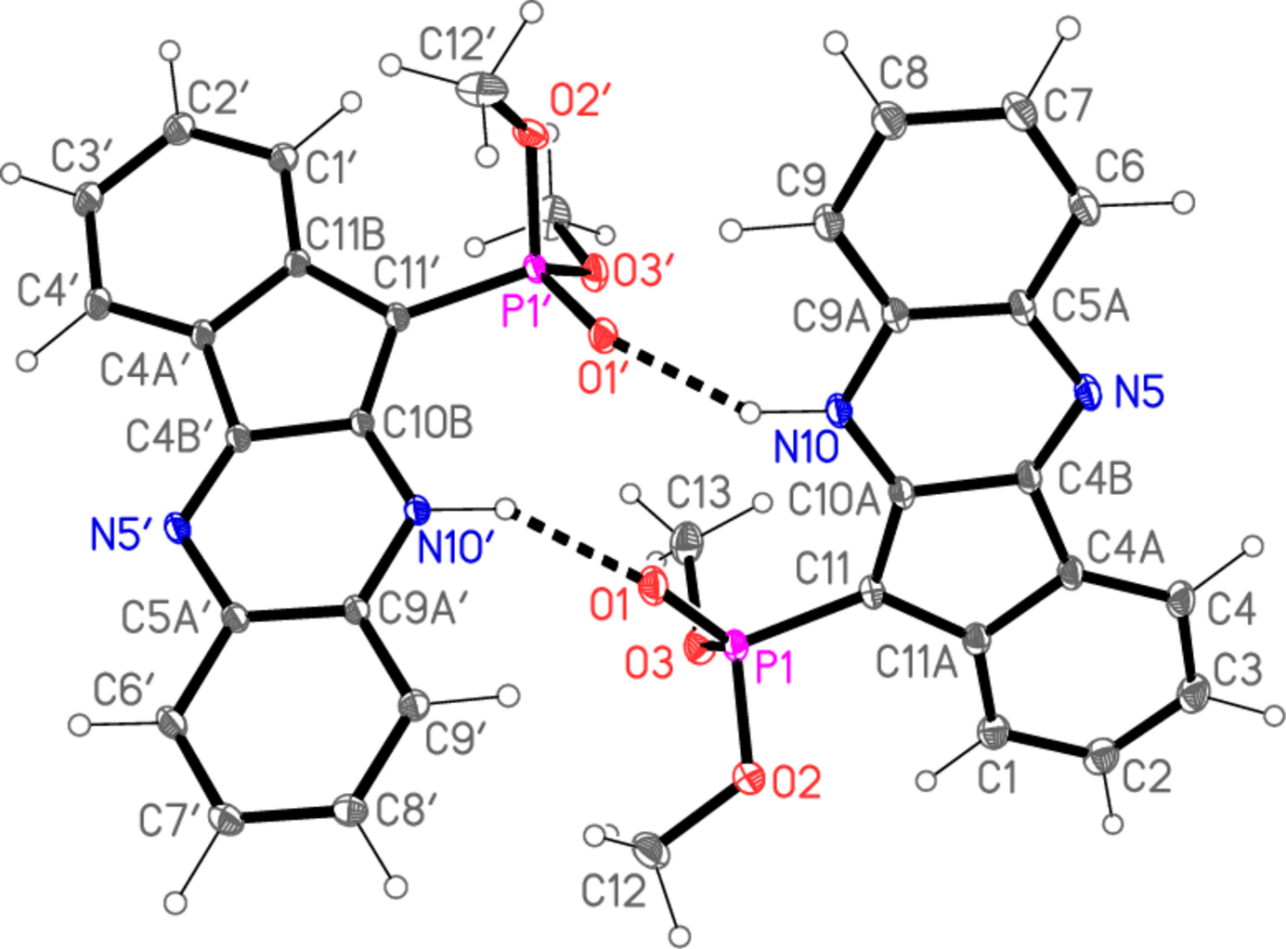
The two independent mol­ecules of compound **4b** in the crystal. Ellipsoids correspond to 50% probability levels. Dashed lines indicate classical hydrogen bonds.

**Table 1:** Selected geometric parameters (Å, °) for **4a**[Chem scheme1]

P1—O1	1.4751 (2)	P1—O3	1.5774 (2)
P1—O2	1.5939 (2)	P1—C11	1.7363 (2)
			
O2—P1—O1	113.967 (9)	C4*B*—C4*A*—C4	131.269 (18)
O3—P1—O1	115.527 (9)	N5—C4*B*—C4*A*	128.493 (17)
O3—P1—O2	99.949 (8)	C11—C10*A*—N10	133.288 (16)
C11—P1—O1	113.500 (9)	C10*A*—C11—P1	126.341 (14)
C11—P1—O2	108.117 (9)	C11*A*—C11—P1	126.227 (14)
C11—P1—O3	104.511 (9)	C11—C11*A*—C1	130.834 (17)
			
O1—P1—C11—C10*A*	18.531 (13)	O3—P1—C11—C10*A*	−108.191 (13)
O1—P1—C11—C11*A*	−169.636 (14)	O3—P1—C11—C11*A*	63.642 (12)
O2—P1—C11—C10*A*	145.999 (13)	C11—P1—O2—C12	−68.052 (15)
O2—P1—C11—C11*A*	−42.169 (12)	C11—P1—O3—C14	−179.223 (14)

**Table 2:** Selected geometric parameters (Å, °) for **4b**[Chem scheme1]

P1—O1	1.4759 (2)	P1′—O1′	1.4753 (2)
P1—O2	1.5799 (2)	P1′—O2′	1.5874 (2)
P1—O3	1.5875 (2)	P1′—O3′	1.5772 (2)
P1—C11	1.7408 (3)	P1′—C11′	1.7410 (2)
			
O2—P1—O1	115.306 (14)	O2′—P1′—O1′	115.087 (13)
O3—P1—O1	113.594 (13)	O3′—P1′—O1′	109.989 (13)
O3—P1—O2	100.738 (14)	O3′—P1′—O2′	101.136 (13)
C11—P1—O1	112.201 (14)	C11′—P1′—O1′	111.009 (12)
C11—P1—O2	104.519 (13)	C11′—P1′—O2′	107.525 (12)
C11—P1—O3	109.554 (13)	C11′—P1′—O3′	111.741 (13)
C4*B*—C4*A*—C4	130.57 (3)	C4*B*′—C4*A*′—C4′	130.60 (2)
N5—C4*B*—C4*A*	128.81 (2)	N5′—C4*B*′—C4*A*′	129.01 (2)
C11—C10*A*—N10	133.08 (2)	C11′—C10*B*—N10′	132.67 (2)
C10*A*—C11—P1	123.67 (2)	C10*B*—C11′—P1′	122.594 (18)
C11*A*—C11—P1	129.22 (2)	C11*B*—C11′—P1′	130.519 (18)
C11—C11*A*—C1	131.25 (3)	C11′—C11*B*—C1′	131.45 (2)
			
O1—P1—C11—C10*A*	−7.878 (18)	O1′—P1′—C11′—C10*B*	−15.047 (18)
O1—P1—C11—C11*A*	167.15 (2)	O1′—P1′—C11′—C11*B*	160.77 (2)
O2—P1—C11—C10*A*	−133.511 (19)	O2′—P1′—C11′—C10*B*	−141.742 (19)
O2—P1—C11—C11*A*	41.517 (18)	O2′—P1′—C11′—C11*B*	34.073 (18)
O3—P1—C11—C10*A*	119.259 (18)	O3′—P1′—C11′—C10*B*	108.139 (18)
O3—P1—C11—C11*A*	−65.713 (18)	O3′—P1′—C11′—C11*B*	−76.046 (18)
C11—P1—O2—C12	177.92 (2)	C11′—P1′—O2′—C12′	65.76 (2)
C11—P1—O3—C13	−67.20 (2)	C11′—P1′—O3′—C13′	57.67 (2)

**Table 3:** Hydrogen-bond geometry (Å, °) for **4a**[Chem scheme1]

*D*—H⋯*A*	*D*—H	H⋯*A*	*D*⋯*A*	*D*—H⋯*A*
C9—H9⋯O1^i^	1.065 (4)	2.334 (4)	3.2118 (3)	138.7 (3)
N10—H10⋯O1^i^	1.006 (4)	1.990 (4)	2.9324 (2)	154.8 (4)
N10—H10⋯O1	1.006 (4)	2.492 (4)	3.1488 (2)	122.5 (3)

Symmetry code: (i) 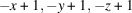.

**Table 4:** Hydrogen-bond geometry (Å, °) for **4b**[Chem scheme1]

*D*—H⋯*A*	*D*—H	H⋯*A*	*D*⋯*A*	*D*—H⋯*A*
C9—H9⋯O1′	1.077 (5)	2.341 (5)	3.1776 (4)	133.3 (4)
N10—H10⋯O1	1.019 (5)	2.343 (6)	2.9896 (3)	120.3 (4)
N10—H10⋯O1′	1.019 (5)	1.911 (6)	2.8468 (3)	151.2 (5)
C9′—H9′⋯O1	1.077 (5)	2.540 (5)	3.2887 (4)	125.8 (4)
N10′—H10′⋯O1	1.015 (6)	1.956 (6)	2.8741 (3)	148.9 (5)
N10′—H10′⋯O1′	1.015 (6)	2.290 (6)	2.9340 (3)	120.1 (4)
C4—H4⋯O3′^i^	1.079 (5)	2.398 (5)	3.2761 (4)	137.5 (4)
C8′—H8′⋯O2^ii^	1.079 (5)	2.416 (5)	3.3475 (4)	143.8 (4)
C13′—H13*e*⋯N5′^iii^	1.075 (6)	2.588 (6)	3.6340 (5)	164.1 (5)

Symmetry codes: (i) 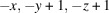; (ii) 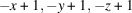; (iii) 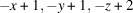.

**Table 5:** Experimental details

	**4a**	**4b**
Crystal data		
Chemical formula	C_19_H_19_N_2_O_3_P	C_17_H_15_N_2_O_3_P
*M*_r_	354.35	326.29
Crystal system, space group	Triclinic, *P* 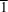	Monoclinic, *P*2_1_/*c*
Temperature (K)	100	100
*a*, *b*, *c* (Å)	7.70923 (7), 10.02049 (8), 12.21059 (10)	14.5034 (2), 13.3068 (2), 16.1730 (2)
α, β, γ (°)	103.0893 (7), 95.7013 (7), 109.1663 (8)	90, 105.2134 (16), 90
*V* (Å^3^)	852.23 (1)	3011.89 (9)
*Z*	2	8
Radiation type	Mo *K*α	Mo *K*α
μ (mm^−1^)	0.18	0.20
Crystal size (mm)	0.20 × 0.20 × 0.10	0.25 × 0.20 × 0.08

Data collection		
Diffractometer	XtaLAB Synergy, HyPix	XtaLAB Synergy, HyPix
Absorption correction	Multi-scan (*CrysAlis PRO*; Rigaku OD, 2024 ▸)	Multi-scan (*CrysAlis PRO*; Rigaku OD, 2024 ▸)
*T*_min_, *T*_max_	0.859, 1.000	0.771, 1.000
No. of measured, independent and observed [*I* ≥ 2u(*I*)] reflections	202119, 20791, 16074	693702, 34614, 24314
*R*_int_	0.043	0.075
θ values (°)	θ_max_ = 53.8, θ_min_ = 2.4	θ_max_ = 52.2, θ_min_ = 2.0
(sin θ/λ)_max_ (Å^−1^)	1.136	1.111

Refinement		
*R*[*F*^2^ > 2σ(*F*^2^)], *wR*(*F*^2^), *S*	0.024, 0.046, 1.08	0.027, 0.048, 1.00
No. of reflections	20791	34614
No. of parameters	397	685
H-atom treatment	All H-atom parameters refined	All H-atom parameters refined
Δρ_max_, Δρ_min_ (e Å^−3^)	0.45, −0.52	0.39, −0.37

Computer programs: *CrysAlis PRO* (Rigaku OD, 2024 ▸), *SHELXT* (Sheldrick, 2015*a* ▸), *OLEX2.refine* (Bourhis *et al.*, 2015 ▸), *XP* (Bruker, 1998 ▸) and *publCIF* (Westrip, 2010 ▸).

## supplementary crystallographic information

### Diethyl (10H-indeno[1,2-b]quinoxalin-11-yl)phosphonate (4a) Crystal data

**Table:**

C_19_H_19_N_2_O_3_P	*Z* = 2
*M**_r_* = 354.35	*F*(000) = 372.436
Triclinic, *P*1	*D*_x_ = 1.381 Mg m^−^^3^
*a* = 7.70923 (7) Å	Mo *K*α radiation, λ = 0.71073 Å
*b* = 10.02049 (8) Å	Cell parameters from 101505 reflections
*c* = 12.21059 (10) Å	θ = 2.9–53.9°
α = 103.0893 (7)°	µ = 0.18 mm^−^^1^
β = 95.7013 (7)°	*T* = 100 K
γ = 109.1663 (8)°	Plate, dark red
*V* = 852.23 (1) Å^3^	0.20 × 0.20 × 0.10 mm

### Diethyl (10H-indeno[1,2-b]quinoxalin-11-yl)phosphonate (4a) Data collection

**Table:**

XtaLAB Synergy, HyPix diffractometer	20791 independent reflections
Radiation source: micro-focus sealed X-ray tube, PhotonJet (Mo) X-ray Source	16074 reflections with *I*≥ 2u(*I*)
Mirror monochromator	*R*_int_ = 0.043
Detector resolution: 10.0000 pixels mm^-1^	θ_max_ = 53.8°, θ_min_ = 2.4°
ω scans	*h* = −16→17
Absorption correction: multi-scan (CrysAlisPro; Rigaku OD, 2024)	*k* = −22→22
*T*_min_ = 0.859, *T*_max_ = 1.000	*l* = −27→27
202119 measured reflections	

### Diethyl (10H-indeno[1,2-b]quinoxalin-11-yl)phosphonate (4a) Refinement

**Table:**

Refinement on *F*^2^	0 constraints
Least-squares matrix: full	Primary atom site location: dual
*R*[*F*^2^ > 2σ(*F*^2^)] = 0.024	All H-atom parameters refined
*wR*(*F*^2^) = 0.046	*w* = 1/[σ^2^(*F*_o_^2^) + (0.0154*P*)^2^ + 0.0072*P*] where *P* = (*F*_o_^2^ + 2*F*_c_^2^)/3
*S* = 1.08	(Δ/σ)_max_ = −0.0002
20791 reflections	Δρ_max_ = 0.45 e Å^−^^3^
397 parameters	Δρ_min_ = −0.52 e Å^−^^3^
0 restraints	

### Diethyl (10H-indeno[1,2-b]quinoxalin-11-yl)phosphonate (4a) Fractional atomic coordinates and isotropic or equivalent isotropic displacement parameters (Å^2^)

**Table:**

	*x*	*y*	*z*	*U*_iso_*/*U*_eq_	
P1	0.735540 (7)	0.518584 (5)	0.697621 (4)	0.009667 (10)	
O1	0.64060 (2)	0.562041 (17)	0.608475 (13)	0.01434 (2)	
O2	0.77859 (2)	0.627165 (16)	0.822847 (12)	0.01349 (2)	
O3	0.93894 (2)	0.522615 (16)	0.685687 (13)	0.01356 (2)	
C1	0.78661 (3)	0.33677 (2)	0.897550 (17)	0.01554 (3)	
H1	0.8791 (6)	0.4515 (5)	0.9219 (3)	0.0292 (10)	
C2	0.79727 (3)	0.24712 (3)	0.968452 (19)	0.01791 (3)	
H2	0.8953 (6)	0.2929 (5)	1.0484 (4)	0.0362 (12)	
C3	0.68200 (3)	0.09830 (3)	0.938199 (19)	0.01788 (3)	
H3	0.6908 (7)	0.0305 (5)	0.9954 (4)	0.0350 (11)	
C4	0.55393 (3)	0.03591 (2)	0.834907 (18)	0.01527 (3)	
H4	0.4646 (6)	−0.0807 (4)	0.8097 (3)	0.0271 (10)	
C4A	0.54350 (3)	0.12458 (2)	0.763644 (16)	0.01177 (3)	
C4B	0.42888 (3)	0.091580 (19)	0.652023 (15)	0.01071 (2)	
N5	0.30773 (2)	−0.033772 (17)	0.589693 (14)	0.01229 (2)	
C5A	0.21844 (3)	−0.033697 (19)	0.485827 (16)	0.01146 (3)	
C6	0.08477 (3)	−0.16696 (2)	0.414946 (18)	0.01492 (3)	
H6	0.0601 (6)	−0.2659 (4)	0.4444 (3)	0.0294 (11)	
C7	−0.00932 (3)	−0.17211 (2)	0.310915 (18)	0.01692 (3)	
H7	−0.1120 (7)	−0.2756 (5)	0.2571 (4)	0.0354 (11)	
C8	0.03031 (3)	−0.04358 (2)	0.274555 (18)	0.01632 (3)	
H8	−0.0435 (6)	−0.0474 (5)	0.1931 (4)	0.0312 (11)	
C9	0.16243 (3)	0.08815 (2)	0.341398 (16)	0.01357 (3)	
H9	0.1964 (6)	0.1860 (5)	0.3143 (3)	0.0305 (10)	
C9A	0.25664 (3)	0.095210 (19)	0.448273 (15)	0.01064 (2)	
N10	0.38733 (2)	0.226579 (17)	0.517304 (13)	0.01071 (2)	
H10	0.4110 (6)	0.3185 (5)	0.4916 (4)	0.0261 (10)	
C10A	0.47786 (3)	0.230470 (19)	0.619053 (15)	0.01008 (2)	
C11	0.61606 (3)	0.341322 (19)	0.704185 (15)	0.01126 (3)	
C11A	0.65814 (3)	0.27543 (2)	0.794171 (16)	0.01169 (3)	
C12	0.62683 (3)	0.65225 (3)	0.87371 (2)	0.01943 (4)	
H12a	0.5805 (7)	0.7222 (6)	0.8331 (4)	0.0443 (13)	
H12b	0.5105 (7)	0.5478 (5)	0.8572 (4)	0.0440 (13)	
C13	0.69775 (5)	0.72245 (5)	0.99964 (2)	0.03473 (7)	
H13a	0.8199 (8)	0.8236 (7)	1.0131 (5)	0.071 (2)	
H13b	0.5894 (8)	0.7459 (7)	1.0412 (4)	0.0627 (18)	
H13c	0.7391 (9)	0.6519 (8)	1.0359 (4)	0.0680 (19)	
C14	1.08332 (3)	0.65507 (2)	0.67902 (2)	0.01778 (4)	
H14a	1.0240 (7)	0.7048 (6)	0.6220 (5)	0.0473 (14)	
H14b	1.1297 (7)	0.7321 (5)	0.7636 (4)	0.0556 (16)	
C15	1.23817 (3)	0.61279 (3)	0.63534 (2)	0.02075 (4)	
H15a	1.1884 (8)	0.5388 (6)	0.5502 (4)	0.0562 (16)	
H15b	1.3508 (6)	0.7078 (5)	0.6312 (4)	0.0443 (13)	
H15c	1.2888 (8)	0.5591 (6)	0.6899 (5)	0.0591 (16)	

### Diethyl (10H-indeno[1,2-b]quinoxalin-11-yl)phosphonate (4a) Atomic displacement parameters (Å^2^)

**Table:**

	*U*^11^	*U*^22^	*U*^33^	*U*^12^	*U*^13^	*U*^23^
P1	0.009523 (18)	0.008755 (16)	0.009357 (17)	0.001951 (13)	0.000711 (13)	0.002368 (12)
O1	0.01583 (6)	0.01372 (5)	0.01289 (5)	0.00462 (4)	−0.00062 (4)	0.00509 (4)
O2	0.01277 (5)	0.01311 (5)	0.01132 (5)	0.00310 (4)	0.00038 (4)	0.00027 (4)
O3	0.01115 (5)	0.01193 (5)	0.01732 (6)	0.00319 (4)	0.00387 (4)	0.00449 (4)
C1	0.01423 (7)	0.01619 (7)	0.01309 (7)	0.00177 (6)	−0.00159 (6)	0.00545 (5)
H1	0.034 (3)	0.027 (2)	0.020 (2)	0.004 (2)	−0.0065 (19)	0.0102 (18)
C2	0.01666 (8)	0.02104 (8)	0.01461 (7)	0.00390 (7)	−0.00116 (6)	0.00834 (6)
H2	0.039 (3)	0.038 (3)	0.026 (2)	0.007 (2)	−0.007 (2)	0.015 (2)
C3	0.01825 (9)	0.02028 (8)	0.01648 (8)	0.00565 (7)	0.00118 (6)	0.01054 (6)
H3	0.040 (3)	0.036 (3)	0.028 (2)	0.010 (2)	−0.004 (2)	0.016 (2)
C4	0.01604 (8)	0.01468 (7)	0.01599 (7)	0.00433 (6)	0.00217 (6)	0.00815 (6)
H4	0.031 (3)	0.024 (2)	0.022 (2)	0.0021 (19)	−0.0001 (19)	0.0110 (18)
C4A	0.01152 (6)	0.01170 (6)	0.01219 (6)	0.00318 (5)	0.00173 (5)	0.00526 (5)
C4B	0.01032 (6)	0.00966 (5)	0.01161 (6)	0.00254 (5)	0.00172 (5)	0.00364 (4)
N5	0.01264 (6)	0.00910 (5)	0.01383 (6)	0.00219 (4)	0.00173 (5)	0.00360 (4)
C5A	0.01103 (6)	0.00917 (5)	0.01227 (6)	0.00224 (5)	0.00165 (5)	0.00164 (4)
C6	0.01498 (8)	0.01037 (6)	0.01519 (7)	0.00142 (5)	0.00133 (6)	0.00083 (5)
H6	0.033 (3)	0.018 (2)	0.025 (2)	−0.0037 (19)	−0.003 (2)	0.0042 (18)
C7	0.01631 (8)	0.01335 (7)	0.01486 (7)	0.00158 (6)	−0.00039 (6)	−0.00097 (5)
H7	0.035 (3)	0.022 (2)	0.037 (3)	0.001 (2)	0.001 (2)	0.004 (2)
C8	0.01627 (8)	0.01535 (7)	0.01277 (7)	0.00362 (6)	−0.00156 (6)	0.00009 (5)
H8	0.035 (3)	0.023 (2)	0.026 (2)	0.004 (2)	−0.0076 (19)	0.0031 (19)
C9	0.01415 (7)	0.01282 (6)	0.01165 (6)	0.00411 (5)	−0.00046 (5)	0.00173 (5)
H9	0.031 (3)	0.030 (2)	0.024 (2)	0.007 (2)	−0.0032 (19)	0.0037 (19)
C9A	0.01021 (6)	0.00995 (5)	0.01056 (6)	0.00304 (5)	0.00114 (5)	0.00177 (4)
N10	0.01066 (6)	0.00955 (5)	0.01079 (5)	0.00258 (4)	0.00077 (4)	0.00278 (4)
H10	0.026 (3)	0.022 (2)	0.025 (2)	0.004 (2)	−0.001 (2)	0.0051 (19)
C10A	0.00945 (6)	0.00917 (5)	0.01049 (6)	0.00206 (4)	0.00111 (5)	0.00285 (4)
C11	0.01079 (6)	0.00980 (5)	0.01114 (6)	0.00129 (5)	0.00026 (5)	0.00337 (4)
C11A	0.01082 (6)	0.01198 (6)	0.01111 (6)	0.00228 (5)	0.00067 (5)	0.00435 (5)
C12	0.01709 (9)	0.02350 (9)	0.01557 (8)	0.00918 (8)	0.00172 (6)	−0.00089 (7)
H12a	0.057 (4)	0.059 (3)	0.038 (3)	0.045 (3)	0.012 (2)	0.017 (2)
H12b	0.031 (3)	0.033 (3)	0.052 (3)	0.001 (2)	0.015 (2)	−0.005 (2)
C13	0.03354 (16)	0.0557 (2)	0.01519 (9)	0.02552 (16)	0.00340 (10)	−0.00324 (11)
H13a	0.029 (3)	0.082 (5)	0.058 (4)	−0.002 (3)	−0.006 (3)	−0.030 (3)
H13b	0.058 (4)	0.093 (5)	0.036 (3)	0.040 (4)	0.016 (3)	−0.005 (3)
H13c	0.093 (5)	0.106 (6)	0.040 (3)	0.068 (5)	0.036 (3)	0.028 (3)
C14	0.01510 (8)	0.01303 (7)	0.02383 (9)	0.00254 (6)	0.00904 (7)	0.00396 (6)
H14a	0.040 (3)	0.052 (3)	0.077 (4)	0.025 (3)	0.028 (3)	0.049 (3)
H14b	0.042 (3)	0.038 (3)	0.050 (3)	−0.017 (2)	0.025 (3)	−0.020 (2)
C15	0.01310 (8)	0.02197 (9)	0.02697 (10)	0.00551 (7)	0.00747 (7)	0.00597 (8)
H15a	0.051 (4)	0.050 (4)	0.046 (3)	0.008 (3)	0.022 (3)	−0.016 (3)
H15b	0.026 (3)	0.032 (3)	0.068 (4)	−0.001 (2)	0.022 (3)	0.011 (3)
H15c	0.036 (3)	0.069 (4)	0.093 (5)	0.024 (3)	0.020 (3)	0.050 (4)

### Diethyl (10H-indeno[1,2-b]quinoxalin-11-yl)phosphonate (4a) Geometric parameters (Å, º)

**Table:**

P1—O1	1.4751 (2)	C10A—C11	1.3890 (2)
P1—O2	1.5939 (1)	C11—C11A	1.4652 (3)
P1—O3	1.5774 (2)	C12—C13	1.4996 (4)
P1—C11	1.7363 (2)	C14—C15	1.4985 (3)
O2—C12	1.4438 (3)	C1—H1	1.088 (4)
O3—C14	1.4506 (3)	C2—H2	1.076 (4)
C1—C2	1.3964 (3)	C3—H3	1.091 (4)
C1—C11A	1.3974 (3)	C4—H4	1.096 (4)
C2—C3	1.4018 (3)	C6—H6	1.095 (4)
C3—C4	1.3952 (3)	C7—H7	1.088 (4)
C4—C4A	1.3915 (3)	C8—H8	1.082 (4)
C4A—C4B	1.4535 (3)	C9—H9	1.065 (4)
C4A—C11A	1.4167 (3)	N10—H10	1.006 (4)
C4B—N5	1.2980 (2)	C12—H12a	1.073 (4)
C4B—C10A	1.4767 (2)	C12—H12b	1.092 (5)
N5—C5A	1.3819 (3)	C13—H13a	1.099 (6)
C5A—C6	1.4106 (3)	C13—H13b	1.081 (5)
C5A—C9A	1.4180 (3)	C13—H13c	1.029 (6)
C6—C7	1.3803 (3)	C14—H14a	1.089 (5)
C7—C8	1.4060 (3)	C14—H14b	1.084 (4)
C8—C9	1.3820 (3)	C15—H15a	1.079 (5)
C9—C9A	1.4042 (3)	C15—H15b	1.073 (4)
C9A—N10	1.3843 (2)	C15—H15c	1.065 (5)
N10—C10A	1.3491 (2)		
			
O2—P1—O1	113.967 (9)	C2—C1—H1	120.3 (2)
O3—P1—O1	115.527 (9)	C11A—C1—H1	120.9 (2)
O3—P1—O2	99.949 (8)	H2—C2—C1	119.6 (2)
C11—P1—O1	113.500 (9)	C3—C2—H2	119.0 (2)
C11—P1—O2	108.117 (9)	H3—C3—C2	120.4 (2)
C11—P1—O3	104.511 (9)	C4—C3—H3	119.4 (2)
C12—O2—P1	119.766 (14)	H4—C4—C3	120.9 (2)
C14—O3—P1	121.223 (14)	C4A—C4—H4	120.6 (2)
C11A—C1—C2	118.749 (19)	H6—C6—C5A	118.0 (2)
C3—C2—C1	121.46 (2)	C7—C6—H6	121.3 (2)
C4—C3—C2	120.207 (19)	H7—C7—C6	119.9 (2)
C4A—C4—C3	118.555 (19)	C8—C7—H7	120.4 (2)
C4B—C4A—C4	131.269 (18)	H8—C8—C7	119.7 (2)
C11A—C4A—C4	121.570 (18)	C9—C8—H8	119.3 (2)
C11A—C4A—C4B	107.158 (16)	H9—C9—C8	122.0 (2)
N5—C4B—C4A	128.493 (17)	C9A—C9—H9	118.2 (2)
C10A—C4B—C4A	106.644 (15)	H10—N10—C9A	119.3 (2)
C10A—C4B—N5	124.856 (17)	C10A—N10—H10	120.9 (2)
C5A—N5—C4B	116.246 (16)	H12a—C12—O2	108.0 (3)
C6—C5A—N5	118.564 (17)	H12b—C12—O2	109.4 (2)
C9A—C5A—N5	122.348 (16)	H12b—C12—H12a	108.3 (4)
C9A—C5A—C6	119.088 (18)	C13—C12—H12a	111.6 (3)
C7—C6—C5A	120.688 (19)	C13—C12—H12b	111.3 (3)
C8—C7—C6	119.637 (18)	H13a—C13—C12	109.5 (3)
C9—C8—C7	120.994 (19)	H13b—C13—C12	110.8 (3)
C9A—C9—C8	119.845 (19)	H13b—C13—H13a	110.6 (5)
C9—C9A—C5A	119.732 (17)	H13c—C13—C12	109.5 (3)
N10—C9A—C5A	119.526 (16)	H13c—C13—H13a	108.2 (5)
N10—C9A—C9	120.742 (17)	H13c—C13—H13b	108.2 (4)
C10A—N10—C9A	119.745 (15)	H14a—C14—O3	109.2 (3)
N10—C10A—C4B	117.271 (15)	H14b—C14—O3	108.3 (3)
C11—C10A—C4B	109.436 (15)	H14b—C14—H14a	107.9 (4)
C11—C10A—N10	133.288 (16)	C15—C14—H14a	111.3 (3)
C10A—C11—P1	126.341 (14)	C15—C14—H14b	112.3 (3)
C11A—C11—P1	126.227 (14)	H15a—C15—C14	110.3 (3)
C11A—C11—C10A	107.036 (15)	H15b—C15—C14	110.8 (3)
C4A—C11A—C1	119.454 (17)	H15b—C15—H15a	107.7 (4)
C11—C11A—C1	130.834 (17)	H15c—C15—C14	110.0 (3)
C11—C11A—C4A	109.711 (16)	H15c—C15—H15a	108.5 (5)
C13—C12—O2	108.18 (2)	H15c—C15—H15b	109.5 (4)
C15—C14—O3	107.830 (18)		
			
P1—O2—C12—C13	164.50 (3)	C4A—C4B—C10A—N10	−179.790 (15)
P1—O3—C14—C15	−164.137 (19)	C4A—C4B—C10A—C11	0.910 (18)
P1—C11—C10A—C4B	172.891 (17)	C4A—C11A—C11—C10A	−0.572 (18)
P1—C11—C10A—N10	−6.25 (2)	C4B—C4A—C11A—C11	1.133 (18)
P1—C11—C11A—C1	6.03 (2)	C4B—N5—C5A—C6	−179.714 (19)
P1—C11—C11A—C4A	−173.698 (18)	C4B—N5—C5A—C9A	0.34 (2)
O1—P1—O2—C12	59.146 (15)	C4B—C10A—N10—C9A	−0.87 (2)
O1—P1—O3—C14	55.324 (14)	C4B—C10A—C11—C11A	−0.224 (17)
O1—P1—C11—C10A	18.531 (13)	N5—C4B—C4A—C11A	177.82 (2)
O1—P1—C11—C11A	−169.636 (14)	N5—C4B—C10A—N10	1.11 (2)
O2—P1—O3—C14	−67.415 (14)	N5—C4B—C10A—C11	−178.19 (2)
O2—P1—C11—C10A	145.999 (13)	N5—C5A—C6—C7	179.40 (2)
O2—P1—C11—C11A	−42.169 (12)	N5—C5A—C9A—C9	179.494 (19)
O3—P1—O2—C12	−177.020 (15)	N5—C5A—C9A—N10	−0.17 (2)
O3—P1—C11—C10A	−108.191 (13)	C5A—N5—C4B—C10A	−0.81 (2)
O3—P1—C11—C11A	63.642 (12)	C5A—C6—C7—C8	0.85 (3)
C1—C2—C3—C4	0.54 (3)	C5A—C9A—C9—C8	1.35 (2)
C1—C11A—C4A—C4	0.81 (2)	C5A—C9A—N10—C10A	0.47 (2)
C1—C11A—C4A—C4B	−178.63 (2)	C6—C5A—C9A—C9	−0.45 (2)
C1—C11A—C11—C10A	179.16 (2)	C6—C5A—C9A—N10	179.885 (18)
C2—C1—C11A—C4A	−0.43 (3)	C6—C7—C8—C9	0.07 (3)
C2—C1—C11A—C11	179.865 (19)	C7—C6—C5A—C9A	−0.66 (2)
C2—C3—C4—C4A	−0.17 (3)	C7—C8—C9—C9A	−1.18 (3)
C3—C2—C1—C11A	−0.23 (3)	C8—C9—C9A—N10	−178.983 (19)
C3—C4—C4A—C4B	178.786 (19)	C9—C9A—N10—C10A	−179.192 (18)
C3—C4—C4A—C11A	−0.50 (3)	C9A—N10—C10A—C11	178.222 (17)
C4—C4A—C4B—N5	−1.55 (3)	N10—C10A—C11—C11A	−179.37 (2)
C4—C4A—C4B—C10A	179.40 (2)	C10A—C4B—C4A—C11A	−1.234 (17)
C4—C4A—C11A—C11	−179.43 (2)	C11—P1—O2—C12	−68.052 (15)
C4A—C4B—N5—C5A	−179.71 (2)	C11—P1—O3—C14	−179.223 (14)

### Diethyl (10H-indeno[1,2-b]quinoxalin-11-yl)phosphonate (4a) Hydrogen-bond geometry (Å, º)

**Table:**

*D*—H···*A*	*D*—H	H···*A*	*D*···*A*	*D*—H···*A*
C9—H9···O1^i^	1.065 (4)	2.334 (4)	3.2118 (3)	138.7 (3)
N10—H10···O1^i^	1.006 (4)	1.990 (4)	2.9324 (2)	154.8 (4)
N10—H10···O1	1.006 (4)	2.492 (4)	3.1488 (2)	122.5 (3)

Symmetry code: (i) −*x*+1, −*y*+1, −*z*+1.

### (4b) Crystal data

**Table:**

C_17_H_15_N_2_O_3_P	*F*(000) = 1361.688
*M**_r_* = 326.29	*D*_x_ = 1.439 Mg m^−^^3^
Monoclinic, *P*2_1_/*c*	Mo *K*α radiation, λ = 0.71073 Å
*a* = 14.5034 (2) Å	Cell parameters from 237034 reflections
*b* = 13.3068 (2) Å	θ = 2.0–54.0°
*c* = 16.1730 (2) Å	µ = 0.20 mm^−^^1^
β = 105.2134 (16)°	*T* = 100 K
*V* = 3011.89 (9) Å^3^	Tablet, black
*Z* = 8	0.25 × 0.20 × 0.08 mm

### (4b) Data collection

**Table:**

XtaLAB Synergy, HyPix diffractometer	34614 independent reflections
Radiation source: micro-focus sealed X-ray tube, PhotonJet (Mo) X-ray Source	24314 reflections with *I*≥ 2u(*I*)
Mirror monochromator	*R*_int_ = 0.075
Detector resolution: 10.0000 pixels mm^-1^	θ_max_ = 52.2°, θ_min_ = 2.0°
ω scans	*h* = −32→32
Absorption correction: multi-scan (CrysAlisPro; Rigaku OD, 2024)	*k* = −30→30
*T*_min_ = 0.771, *T*_max_ = 1.000	*l* = −36→36
693702 measured reflections	

### (4b) Refinement

**Table:**

Refinement on *F*^2^	0 constraints
Least-squares matrix: full	Primary atom site location: intrinsic
*R*[*F*^2^ > 2σ(*F*^2^)] = 0.027	All H-atom parameters refined
*wR*(*F*^2^) = 0.048	*w* = 1/[σ^2^(*F*_o_^2^) + (0.*P*)^2^ + 0.1856*P*] where *P* = (*F*_o_^2^ + 2*F*_c_^2^)/3
*S* = 1.00	(Δ/σ)_max_ = 0.0002
34614 reflections	Δρ_max_ = 0.39 e Å^−^^3^
685 parameters	Δρ_min_ = −0.37 e Å^−^^3^
0 restraints	

### (4b) Fractional atomic coordinates and isotropic or equivalent isotropic displacement parameters (Å^2^)

**Table:**

	*x*	*y*	*z*	*U*_iso_*/*U*_eq_	
P1	0.317518 (5)	0.440073 (6)	0.579310 (4)	0.012615 (12)	
O1	0.357457 (16)	0.528546 (18)	0.631540 (15)	0.01828 (4)	
O2	0.379393 (16)	0.39866 (2)	0.519608 (14)	0.01901 (4)	
O3	0.311029 (18)	0.342916 (18)	0.634466 (14)	0.01809 (4)	
C1	0.16810 (2)	0.31023 (2)	0.406652 (19)	0.01643 (4)	
H1	0.2299 (4)	0.2663 (4)	0.4390 (4)	0.0320 (14)	
C2	0.10319 (2)	0.27303 (3)	0.33330 (2)	0.01895 (5)	
H2	0.1155 (4)	0.1995 (5)	0.3098 (4)	0.0367 (15)	
C3	0.02212 (2)	0.32778 (3)	0.28992 (2)	0.01962 (5)	
H3	−0.0262 (4)	0.2963 (5)	0.2337 (4)	0.0380 (15)	
C4	0.00421 (2)	0.42200 (3)	0.320078 (19)	0.01736 (5)	
H4	−0.0583 (4)	0.4649 (4)	0.2887 (3)	0.0343 (14)	
C4A	0.068723 (19)	0.45957 (2)	0.393133 (17)	0.01369 (4)	
C4B	0.069075 (18)	0.55395 (2)	0.438429 (16)	0.01282 (4)	
N5	0.009096 (17)	0.62820 (2)	0.419749 (15)	0.01456 (4)	
C5A	0.028362 (18)	0.71039 (2)	0.474052 (17)	0.01339 (4)	
C6	−0.03451 (2)	0.79292 (2)	0.456706 (19)	0.01631 (4)	
H6	−0.0949 (4)	0.7889 (4)	0.4007 (3)	0.0290 (13)	
C7	−0.01921 (2)	0.87553 (2)	0.51019 (2)	0.01710 (5)	
H7	−0.0679 (4)	0.9384 (4)	0.4975 (4)	0.0357 (15)	
C8	0.06004 (2)	0.87813 (2)	0.582367 (19)	0.01628 (4)	
H8	0.0708 (4)	0.9430 (4)	0.6248 (3)	0.0322 (14)	
C9	0.12373 (2)	0.79882 (2)	0.600438 (18)	0.01461 (4)	
H9	0.1843 (4)	0.7986 (4)	0.6558 (3)	0.0311 (14)	
C9A	0.108338 (18)	0.71398 (2)	0.546586 (16)	0.01255 (4)	
N10	0.170779 (16)	0.633742 (19)	0.563476 (14)	0.01292 (3)	
H10	0.2253 (4)	0.6345 (5)	0.6181 (4)	0.0260 (14)	
C10A	0.155395 (18)	0.55287 (2)	0.511226 (16)	0.01205 (4)	
C11	0.206092 (18)	0.46473 (2)	0.509758 (16)	0.01292 (4)	
C11A	0.151144 (19)	0.40507 (2)	0.436858 (17)	0.01319 (4)	
C12	0.47810 (2)	0.37306 (3)	0.55501 (3)	0.02266 (6)	
H12a	0.5025 (5)	0.3367 (6)	0.5066 (5)	0.062 (2)	
H12b	0.5191 (5)	0.4385 (6)	0.5772 (6)	0.072 (2)	
H12c	0.4842 (5)	0.3227 (6)	0.6061 (5)	0.066 (2)	
C13	0.25410 (3)	0.35041 (3)	0.69527 (2)	0.02198 (6)	
H13a	0.1795 (4)	0.3607 (5)	0.6614 (4)	0.0442 (17)	
H13b	0.2651 (5)	0.2814 (5)	0.7315 (4)	0.0508 (18)	
H13c	0.2776 (5)	0.4135 (5)	0.7374 (4)	0.0423 (16)	
P1'	0.268612 (5)	0.652406 (6)	0.823381 (4)	0.011011 (11)	
O1'	0.276939 (15)	0.670152 (19)	0.735586 (12)	0.01603 (4)	
O2'	0.215163 (15)	0.738239 (18)	0.860873 (14)	0.01619 (4)	
O3'	0.201161 (15)	0.559670 (19)	0.824289 (14)	0.01654 (4)	
C1'	0.36046 (2)	0.66872 (2)	1.048246 (17)	0.01411 (4)	
H1'	0.2849 (4)	0.6837 (5)	1.0285 (3)	0.0303 (13)	
C2'	0.40963 (2)	0.67425 (3)	1.134825 (17)	0.01673 (5)	
H2'	0.3707 (4)	0.6935 (5)	1.1808 (3)	0.0365 (15)	
C3'	0.50824 (2)	0.65629 (3)	1.162799 (17)	0.01744 (5)	
H3'	0.5438 (4)	0.6614 (5)	1.2302 (3)	0.0382 (16)	
C4'	0.55989 (2)	0.63357 (2)	1.103478 (16)	0.01481 (4)	
H4'	0.6371 (4)	0.6203 (5)	1.1231 (3)	0.0323 (14)	
C4A'	0.511209 (18)	0.62819 (2)	1.017174 (15)	0.01130 (3)	
C4B'	0.546213 (17)	0.61130 (2)	0.942085 (15)	0.01077 (3)	
N5'	0.633263 (16)	0.596063 (19)	0.937569 (14)	0.01234 (3)	
C5A'	0.645771 (17)	0.58620 (2)	0.856098 (16)	0.01150 (4)	
C6'	0.738750 (19)	0.57064 (2)	0.847018 (18)	0.01499 (4)	
H6'	0.7958 (4)	0.5672 (5)	0.9052 (3)	0.0311 (14)	
C7'	0.75505 (2)	0.56254 (3)	0.766930 (19)	0.01659 (4)	
H7'	0.8266 (4)	0.5527 (5)	0.7599 (3)	0.0343 (14)	
C8'	0.67786 (2)	0.56988 (2)	0.693158 (18)	0.01587 (4)	
H8'	0.6900 (4)	0.5654 (5)	0.6303 (3)	0.0321 (14)	
C9'	0.585754 (19)	0.58428 (2)	0.700032 (17)	0.01377 (4)	
H9'	0.5260 (4)	0.5913 (5)	0.6444 (3)	0.0283 (13)	
C9A'	0.568756 (17)	0.59165 (2)	0.781465 (15)	0.01102 (3)	
N10'	0.477300 (15)	0.605766 (19)	0.789321 (13)	0.01154 (3)	
H10'	0.4216 (4)	0.6008 (5)	0.7362 (4)	0.0254 (14)	
C10B	0.462305 (17)	0.61726 (2)	0.867484 (15)	0.01035 (3)	
C11'	0.380655 (17)	0.63619 (2)	0.894584 (15)	0.01104 (3)	
C11B	0.411190 (17)	0.64470 (2)	0.988366 (15)	0.01097 (3)	
C12'	0.25458 (3)	0.83824 (3)	0.86579 (3)	0.02425 (6)	
H12d	0.2970 (5)	0.8477 (5)	0.8210 (5)	0.055 (2)	
H12e	0.1961 (5)	0.8892 (5)	0.8511 (5)	0.057 (2)	
H12f	0.2976 (6)	0.8519 (5)	0.9301 (4)	0.063 (2)	
C13'	0.17800 (3)	0.52608 (3)	0.90090 (2)	0.02312 (6)	
H13d	0.1315 (5)	0.4631 (5)	0.8820 (4)	0.059 (2)	
H13e	0.2415 (4)	0.5032 (6)	0.9481 (4)	0.0508 (19)	
H13f	0.1426 (5)	0.5844 (5)	0.9258 (4)	0.0511 (18)	

### (4b) Atomic displacement parameters (Å^2^)

**Table:**

	*U*^11^	*U*^22^	*U*^33^	*U*^12^	*U*^13^	*U*^23^
P1	0.01106 (3)	0.01406 (3)	0.01087 (2)	0.00155 (2)	−0.000413 (19)	0.00007 (2)
O1	0.01445 (8)	0.01772 (10)	0.01903 (9)	−0.00011 (7)	−0.00208 (7)	−0.00379 (7)
O2	0.01441 (8)	0.02644 (12)	0.01518 (8)	0.00453 (8)	0.00213 (6)	−0.00156 (7)
O3	0.02133 (10)	0.01628 (9)	0.01463 (8)	0.00304 (7)	0.00110 (7)	0.00312 (7)
C1	0.01576 (10)	0.01608 (11)	0.01700 (10)	−0.00204 (9)	0.00350 (8)	−0.00151 (8)
H1	0.032 (3)	0.025 (3)	0.035 (3)	0.004 (3)	0.002 (3)	−0.005 (3)
C2	0.01862 (12)	0.01875 (13)	0.01919 (12)	−0.00493 (10)	0.00446 (9)	−0.00430 (9)
H2	0.031 (3)	0.038 (4)	0.040 (4)	−0.007 (3)	0.008 (3)	−0.013 (3)
C3	0.01651 (11)	0.02360 (14)	0.01736 (11)	−0.00617 (10)	0.00196 (9)	−0.00521 (10)
H3	0.028 (3)	0.048 (4)	0.035 (3)	−0.006 (3)	0.003 (3)	−0.015 (3)
C4	0.01202 (10)	0.02307 (13)	0.01480 (10)	−0.00366 (9)	−0.00036 (8)	−0.00285 (9)
H4	0.021 (3)	0.041 (4)	0.034 (3)	0.004 (3)	−0.007 (2)	−0.005 (3)
C4A	0.00998 (8)	0.01757 (11)	0.01216 (9)	−0.00207 (7)	0.00048 (7)	−0.00069 (7)
C4B	0.00912 (8)	0.01649 (11)	0.01141 (8)	−0.00051 (7)	0.00013 (6)	0.00057 (7)
N5	0.00955 (7)	0.01852 (10)	0.01328 (8)	0.00084 (7)	−0.00116 (6)	0.00055 (7)
C5A	0.00941 (8)	0.01619 (11)	0.01293 (9)	0.00089 (7)	0.00001 (7)	0.00176 (7)
C6	0.01118 (9)	0.01770 (12)	0.01748 (10)	0.00227 (8)	−0.00078 (8)	0.00172 (9)
H6	0.019 (3)	0.031 (3)	0.031 (3)	0.010 (3)	−0.004 (2)	−0.002 (3)
C7	0.01316 (10)	0.01661 (12)	0.01961 (11)	0.00278 (8)	0.00092 (8)	0.00186 (9)
H7	0.030 (3)	0.032 (4)	0.040 (4)	0.010 (3)	0.001 (3)	−0.004 (3)
C8	0.01457 (10)	0.01554 (11)	0.01723 (10)	0.00146 (8)	0.00151 (8)	0.00066 (8)
H8	0.034 (3)	0.025 (3)	0.034 (3)	0.004 (3)	0.003 (3)	−0.005 (3)
C9	0.01300 (9)	0.01552 (11)	0.01344 (9)	0.00080 (8)	0.00015 (7)	0.00092 (8)
H9	0.028 (3)	0.031 (3)	0.029 (3)	0.005 (3)	−0.002 (3)	0.000 (3)
C9A	0.01000 (8)	0.01473 (10)	0.01161 (8)	0.00051 (7)	0.00049 (6)	0.00167 (7)
N10	0.01041 (7)	0.01511 (9)	0.01119 (7)	0.00090 (6)	−0.00079 (6)	0.00073 (6)
H10	0.019 (3)	0.028 (4)	0.025 (3)	0.002 (3)	−0.005 (3)	−0.002 (3)
C10A	0.00941 (8)	0.01462 (10)	0.01069 (8)	0.00008 (7)	0.00009 (6)	0.00098 (7)
C11	0.01050 (8)	0.01473 (10)	0.01182 (8)	0.00021 (7)	−0.00013 (7)	0.00064 (7)
C11A	0.01102 (9)	0.01541 (11)	0.01235 (9)	−0.00177 (7)	0.00166 (7)	0.00013 (7)
C12	0.01573 (12)	0.02167 (15)	0.02833 (15)	0.00570 (10)	0.00176 (10)	−0.00500 (12)
H12a	0.049 (4)	0.068 (5)	0.070 (5)	0.024 (4)	0.017 (4)	−0.026 (4)
H12b	0.030 (4)	0.046 (5)	0.129 (7)	−0.001 (3)	0.001 (4)	−0.035 (5)
H12c	0.047 (4)	0.092 (6)	0.059 (5)	0.022 (4)	0.016 (4)	0.021 (4)
C13	0.02229 (13)	0.02639 (16)	0.01568 (11)	−0.00236 (12)	0.00216 (10)	0.00386 (11)
H13a	0.028 (3)	0.072 (5)	0.030 (3)	0.000 (3)	0.001 (3)	0.005 (3)
H13b	0.064 (5)	0.049 (4)	0.043 (4)	0.008 (4)	0.021 (4)	0.018 (3)
H13c	0.051 (4)	0.045 (4)	0.034 (3)	−0.011 (3)	0.014 (3)	−0.013 (3)
P1'	0.00780 (2)	0.01581 (3)	0.00883 (2)	0.00101 (2)	0.001129 (17)	0.00069 (2)
O1'	0.01295 (7)	0.02523 (11)	0.00931 (7)	0.00256 (7)	0.00187 (6)	0.00229 (6)
O2'	0.01231 (7)	0.01987 (9)	0.01747 (8)	0.00380 (7)	0.00583 (6)	0.00069 (7)
O3'	0.01202 (7)	0.02125 (10)	0.01487 (8)	−0.00406 (7)	0.00091 (6)	−0.00061 (7)
C1'	0.01249 (9)	0.01939 (12)	0.01075 (8)	0.00092 (8)	0.00358 (7)	−0.00114 (8)
H1'	0.024 (3)	0.044 (4)	0.023 (3)	0.008 (3)	0.006 (2)	0.000 (3)
C2'	0.01668 (11)	0.02349 (13)	0.01031 (9)	0.00071 (9)	0.00406 (8)	−0.00211 (8)
H2'	0.033 (3)	0.054 (4)	0.025 (3)	0.005 (3)	0.012 (3)	−0.007 (3)
C3'	0.01707 (11)	0.02531 (14)	0.00901 (8)	0.00080 (10)	0.00176 (8)	−0.00179 (8)
H3'	0.032 (3)	0.063 (5)	0.019 (3)	0.000 (3)	0.005 (2)	0.000 (3)
C4'	0.01273 (9)	0.02148 (12)	0.00875 (8)	0.00091 (8)	0.00020 (7)	−0.00056 (8)
H4'	0.018 (3)	0.053 (4)	0.022 (3)	0.006 (3)	0.000 (2)	−0.002 (3)
C4A'	0.01016 (8)	0.01477 (10)	0.00806 (7)	0.00028 (7)	0.00076 (6)	−0.00006 (7)
C4B'	0.00863 (8)	0.01410 (10)	0.00876 (7)	0.00056 (7)	0.00082 (6)	0.00035 (6)
N5'	0.00836 (7)	0.01752 (9)	0.01015 (7)	0.00078 (6)	0.00071 (6)	0.00061 (6)
C5A'	0.00823 (8)	0.01484 (10)	0.01105 (8)	0.00023 (7)	0.00188 (6)	0.00040 (7)
C6'	0.00850 (8)	0.02167 (12)	0.01458 (9)	0.00051 (8)	0.00266 (7)	0.00037 (8)
H6'	0.016 (3)	0.050 (4)	0.026 (3)	0.003 (3)	0.002 (2)	0.001 (3)
C7'	0.01056 (9)	0.02331 (13)	0.01698 (10)	0.00015 (9)	0.00553 (8)	−0.00056 (9)
H7'	0.022 (3)	0.051 (4)	0.030 (3)	0.002 (3)	0.008 (2)	0.000 (3)
C8'	0.01292 (9)	0.02190 (13)	0.01415 (9)	−0.00079 (9)	0.00598 (8)	−0.00117 (8)
H8'	0.020 (3)	0.055 (4)	0.023 (3)	−0.003 (3)	0.008 (2)	−0.002 (3)
C9'	0.01154 (9)	0.01937 (11)	0.01073 (8)	−0.00054 (8)	0.00353 (7)	−0.00067 (8)
H9'	0.021 (3)	0.046 (4)	0.019 (3)	0.000 (3)	0.008 (2)	−0.002 (3)
C9A'	0.00900 (8)	0.01410 (10)	0.00978 (8)	−0.00003 (7)	0.00212 (6)	−0.00018 (7)
N10'	0.00847 (7)	0.01699 (9)	0.00858 (7)	0.00052 (6)	0.00122 (5)	−0.00002 (6)
H10'	0.015 (3)	0.038 (4)	0.021 (3)	0.004 (3)	0.002 (2)	0.001 (3)
C10B	0.00818 (7)	0.01393 (9)	0.00842 (7)	0.00047 (6)	0.00124 (6)	0.00010 (6)
C11'	0.00828 (7)	0.01563 (10)	0.00864 (7)	0.00065 (7)	0.00121 (6)	−0.00003 (7)
C11B	0.00983 (8)	0.01403 (10)	0.00865 (7)	0.00020 (7)	0.00172 (6)	−0.00017 (7)
C12'	0.02597 (15)	0.01829 (14)	0.03219 (17)	0.00363 (11)	0.01419 (13)	−0.00146 (12)
H12d	0.072 (5)	0.029 (4)	0.084 (5)	−0.009 (3)	0.058 (4)	−0.009 (3)
H12e	0.049 (4)	0.040 (4)	0.088 (6)	0.017 (3)	0.025 (4)	0.012 (4)
H12f	0.080 (6)	0.050 (5)	0.047 (4)	−0.016 (4)	−0.006 (4)	−0.016 (4)
C13'	0.02234 (14)	0.02669 (16)	0.02059 (13)	−0.00785 (12)	0.00607 (11)	0.00277 (11)
H13d	0.067 (5)	0.064 (5)	0.047 (4)	−0.039 (4)	0.018 (4)	0.000 (4)
H13e	0.038 (4)	0.070 (5)	0.037 (4)	−0.001 (4)	−0.002 (3)	0.024 (4)
H13f	0.060 (5)	0.050 (4)	0.055 (4)	0.002 (4)	0.037 (4)	0.004 (3)

### (4b) Geometric parameters (Å, º)

**Table:**

P1—O1	1.4759 (2)	N5'—C5A'	1.3826 (3)
P1—O2	1.5799 (2)	C5A'—C6'	1.4095 (4)
P1—O3	1.5875 (2)	C5A'—C9A'	1.4150 (3)
P1—C11	1.7408 (3)	C6'—C7'	1.3806 (4)
O2—C12	1.4363 (4)	C7'—C8'	1.4091 (4)
O3—C13	1.4440 (4)	C8'—C9'	1.3825 (4)
C1—C2	1.3973 (4)	C9'—C9A'	1.4055 (3)
C1—C11A	1.3982 (4)	C9A'—N10'	1.3781 (3)
C2—C3	1.4045 (5)	N10'—C10B	1.3465 (3)
C3—C4	1.3942 (5)	C10B—C11'	1.3894 (3)
C4—C4A	1.3933 (4)	C11'—C11B	1.4686 (3)
C4A—C4B	1.4532 (4)	C1—H1	1.083 (5)
C4A—C11A	1.4182 (4)	C2—H2	1.082 (6)
C4B—N5	1.2986 (4)	C3—H3	1.077 (5)
C4B—C10A	1.4765 (3)	C4—H4	1.079 (5)
N5—C5A	1.3844 (4)	C6—H6	1.084 (5)
C5A—C6	1.4079 (4)	C7—H7	1.080 (5)
C5A—C9A	1.4176 (4)	C8—H8	1.088 (5)
C6—C7	1.3802 (5)	C9—H9	1.077 (5)
C7—C8	1.4073 (4)	N10—H10	1.019 (5)
C8—C9	1.3824 (4)	C12—H12a	1.057 (6)
C9—C9A	1.4074 (4)	C12—H12b	1.062 (7)
C9A—N10	1.3802 (4)	C12—H12c	1.049 (7)
N10—C10A	1.3502 (4)	C13—H13a	1.085 (6)
C10A—C11	1.3878 (4)	C13—H13b	1.079 (6)
C11—C11A	1.4704 (4)	C13—H13c	1.079 (6)
P1'—O1'	1.4753 (2)	C1'—H1'	1.076 (5)
P1'—O2'	1.5874 (2)	C2'—H2'	1.076 (5)
P1'—O3'	1.5772 (2)	C3'—H3'	1.078 (5)
P1'—C11'	1.7410 (2)	C4'—H4'	1.095 (5)
O2'—C12'	1.4423 (5)	C6'—H6'	1.078 (5)
O3'—C13'	1.4375 (4)	C7'—H7'	1.081 (5)
C1'—C2'	1.3966 (4)	C8'—H8'	1.079 (5)
C1'—C11B	1.3984 (4)	C9'—H9'	1.077 (5)
C2'—C3'	1.4026 (4)	N10'—H10'	1.016 (6)
C3'—C4'	1.3965 (4)	C12'—H12d	1.074 (6)
C4'—C4A'	1.3909 (3)	C12'—H12e	1.062 (6)
C4A'—C4B'	1.4514 (3)	C12'—H12f	1.079 (7)
C4A'—C11B	1.4195 (3)	C13'—H13d	1.069 (6)
C4B'—N5'	1.2995 (3)	C13'—H13e	1.075 (6)
C4B'—C10B	1.4739 (3)	C13'—H13f	1.066 (7)
			
O2—P1—O1	115.306 (14)	N10'—C10B—C4B'	117.41 (2)
O3—P1—O1	113.594 (13)	C11'—C10B—C4B'	109.91 (2)
O3—P1—O2	100.738 (14)	C11'—C10B—N10'	132.67 (2)
C11—P1—O1	112.201 (14)	C10B—C11'—P1'	122.594 (18)
C11—P1—O2	104.519 (13)	C11B—C11'—P1'	130.519 (18)
C11—P1—O3	109.554 (13)	C11B—C11'—C10B	106.79 (2)
C12—O2—P1	120.38 (2)	C4A'—C11B—C1'	119.09 (2)
C13—O3—P1	117.08 (2)	C11'—C11B—C1'	131.45 (2)
C11A—C1—C2	118.38 (3)	C11'—C11B—C4A'	109.41 (2)
C3—C2—C1	121.98 (3)	C2—C1—H1	120.4 (3)
C4—C3—C2	120.01 (3)	C11A—C1—H1	121.2 (3)
C4A—C4—C3	118.30 (3)	H2—C2—C1	118.8 (3)
C4B—C4A—C4	130.57 (3)	C3—C2—H2	119.2 (3)
C11A—C4A—C4	122.00 (3)	H3—C3—C2	119.6 (3)
C11A—C4A—C4B	107.43 (2)	C4—C3—H3	120.4 (3)
N5—C4B—C4A	128.81 (2)	H4—C4—C3	121.5 (3)
C10A—C4B—C4A	106.48 (2)	C4A—C4—H4	120.2 (3)
C10A—C4B—N5	124.68 (3)	H6—C6—C5A	117.9 (3)
C5A—N5—C4B	116.24 (2)	C7—C6—H6	121.7 (3)
C6—C5A—N5	118.52 (2)	H7—C7—C6	120.5 (3)
C9A—C5A—N5	122.46 (2)	C8—C7—H7	119.4 (3)
C9A—C5A—C6	119.02 (3)	H8—C8—C7	119.6 (3)
C7—C6—C5A	120.48 (3)	C9—C8—H8	119.7 (3)
C8—C7—C6	120.10 (3)	H9—C9—C8	122.1 (3)
C9—C8—C7	120.77 (3)	C9A—C9—H9	118.3 (3)
C9A—C9—C8	119.53 (3)	H10—N10—C9A	118.9 (3)
C9—C9A—C5A	120.10 (3)	C10A—N10—H10	120.9 (3)
N10—C9A—C5A	119.31 (3)	H12a—C12—O2	107.8 (4)
N10—C9A—C9	120.58 (2)	H12b—C12—O2	110.5 (4)
C10A—N10—C9A	120.04 (2)	H12b—C12—H12a	111.0 (6)
N10—C10A—C4B	117.23 (2)	H12c—C12—O2	109.6 (4)
C11—C10A—C4B	109.68 (2)	H12c—C12—H12a	108.4 (6)
C11—C10A—N10	133.08 (2)	H12c—C12—H12b	109.5 (7)
C10A—C11—P1	123.67 (2)	H13a—C13—O3	109.7 (3)
C11A—C11—P1	129.22 (2)	H13b—C13—O3	106.4 (3)
C11A—C11—C10A	106.97 (2)	H13b—C13—H13a	111.5 (5)
C4A—C11A—C1	119.33 (3)	H13c—C13—O3	110.0 (3)
C11—C11A—C1	131.25 (3)	H13c—C13—H13a	109.1 (5)
C11—C11A—C4A	109.41 (2)	H13c—C13—H13b	110.1 (5)
O2'—P1'—O1'	115.087 (13)	C2'—C1'—H1'	120.0 (3)
O3'—P1'—O1'	109.989 (13)	C11B—C1'—H1'	121.1 (3)
O3'—P1'—O2'	101.136 (13)	H2'—C2'—C1'	118.9 (3)
C11'—P1'—O1'	111.009 (12)	C3'—C2'—H2'	119.6 (3)
C11'—P1'—O2'	107.525 (12)	H3'—C3'—C2'	119.4 (3)
C11'—P1'—O3'	111.741 (13)	C4'—C3'—H3'	120.5 (3)
C12'—O2'—P1'	117.30 (2)	H4'—C4'—C3'	121.9 (3)
C13'—O3'—P1'	122.49 (2)	C4A'—C4'—H4'	119.6 (3)
C11B—C1'—C2'	118.90 (3)	H6'—C6'—C5A'	116.9 (3)
C3'—C2'—C1'	121.57 (3)	C7'—C6'—H6'	122.2 (3)
C4'—C3'—C2'	120.04 (2)	H7'—C7'—C6'	120.9 (3)
C4A'—C4'—C3'	118.56 (3)	C8'—C7'—H7'	119.4 (3)
C4B'—C4A'—C4'	130.60 (2)	H8'—C8'—C7'	120.3 (3)
C11B—C4A'—C4'	121.82 (2)	C9'—C8'—H8'	119.0 (3)
C11B—C4A'—C4B'	107.52 (2)	H9'—C9'—C8'	121.8 (3)
N5'—C4B'—C4A'	129.01 (2)	C9A'—C9'—H9'	118.5 (3)
C10B—C4B'—C4A'	106.36 (2)	H10'—N10'—C9A'	118.9 (3)
C10B—C4B'—N5'	124.61 (2)	C10B—N10'—H10'	120.9 (3)
C5A'—N5'—C4B'	116.15 (2)	H12d—C12'—O2'	111.1 (3)
C6'—C5A'—N5'	118.79 (2)	H12e—C12'—O2'	107.2 (4)
C9A'—C5A'—N5'	122.44 (2)	H12e—C12'—H12d	109.8 (5)
C9A'—C5A'—C6'	118.77 (2)	H12f—C12'—O2'	109.2 (4)
C7'—C6'—C5A'	120.88 (3)	H12f—C12'—H12d	110.1 (6)
C8'—C7'—C6'	119.69 (2)	H12f—C12'—H12e	109.5 (6)
C9'—C8'—C7'	120.75 (2)	H13d—C13'—O3'	105.7 (3)
C9A'—C9'—C8'	119.72 (2)	H13e—C13'—O3'	110.5 (3)
C9'—C9A'—C5A'	120.16 (2)	H13e—C13'—H13d	109.9 (6)
N10'—C9A'—C5A'	119.46 (2)	H13f—C13'—O3'	110.1 (3)
N10'—C9A'—C9'	120.37 (2)	H13f—C13'—H13d	110.4 (6)
C10B—N10'—C9A'	119.90 (2)	H13f—C13'—H13e	110.2 (5)
			
P1—C11—C10A—C4B	174.08 (2)	P1'—C11'—C10B—C4B'	177.69 (2)
P1—C11—C10A—N10	−4.76 (3)	P1'—C11'—C10B—N10'	−1.08 (3)
P1—C11—C11A—C1	5.42 (3)	P1'—C11'—C11B—C1'	−0.47 (3)
P1—C11—C11A—C4A	−173.86 (3)	P1'—C11'—C11B—C4A'	−177.79 (3)
O1—P1—O2—C12	54.27 (2)	O1'—P1'—O2'—C12'	−58.50 (2)
O1—P1—O3—C13	59.15 (2)	O1'—P1'—O3'—C13'	−178.56 (2)
O1—P1—C11—C10A	−7.878 (18)	O1'—P1'—C11'—C10B	−15.047 (18)
O1—P1—C11—C11A	167.15 (2)	O1'—P1'—C11'—C11B	160.77 (2)
O2—P1—O3—C13	−176.97 (2)	O2'—P1'—O3'—C13'	−56.46 (2)
O2—P1—C11—C10A	−133.511 (19)	O2'—P1'—C11'—C10B	−141.742 (19)
O2—P1—C11—C11A	41.517 (18)	O2'—P1'—C11'—C11B	34.073 (18)
O3—P1—O2—C12	−68.44 (2)	O3'—P1'—O2'—C12'	−176.98 (2)
O3—P1—C11—C10A	119.259 (18)	O3'—P1'—C11'—C10B	108.139 (18)
O3—P1—C11—C11A	−65.713 (18)	O3'—P1'—C11'—C11B	−76.046 (18)
C1—C2—C3—C4	−0.36 (4)	C1'—C2'—C3'—C4'	0.88 (4)
C1—C11A—C4A—C4	−0.71 (3)	C1'—C11B—C4A'—C4'	1.11 (3)
C1—C11A—C4A—C4B	179.62 (3)	C1'—C11B—C4A'—C4B'	−176.37 (3)
C1—C11A—C11—C10A	−178.90 (3)	C1'—C11B—C11'—C10B	175.85 (3)
C2—C1—C11A—C4A	0.76 (3)	C2'—C1'—C11B—C4A'	−0.95 (3)
C2—C1—C11A—C11	−178.47 (2)	C2'—C1'—C11B—C11'	−178.05 (3)
C2—C3—C4—C4A	0.42 (4)	C2'—C3'—C4'—C4A'	−0.73 (4)
C3—C2—C1—C11A	−0.24 (4)	C3'—C2'—C1'—C11B	−0.02 (4)
C3—C4—C4A—C4B	179.69 (3)	C3'—C4'—C4A'—C4B'	176.57 (3)
C3—C4—C4A—C11A	0.10 (3)	C3'—C4'—C4A'—C11B	−0.25 (4)
C4—C4A—C4B—N5	−1.90 (4)	C4'—C4A'—C4B'—N5'	0.61 (4)
C4—C4A—C4B—C10A	−179.78 (3)	C4'—C4A'—C4B'—C10B	−177.87 (3)
C4—C4A—C11A—C11	178.67 (3)	C4'—C4A'—C11B—C11'	178.80 (3)
C4A—C4B—N5—C5A	−178.80 (3)	C4A'—C4B'—N5'—C5A'	−177.46 (3)
C4A—C4B—C10A—N10	−179.65 (2)	C4A'—C4B'—C10B—N10'	178.76 (2)
C4A—C4B—C10A—C11	1.31 (2)	C4A'—C4B'—C10B—C11'	−0.22 (2)
C4A—C11A—C11—C10A	1.81 (2)	C4A'—C11B—C11'—C10B	−1.47 (2)
C4B—C4A—C11A—C11	−1.00 (2)	C4B'—C4A'—C11B—C11'	1.33 (2)
C4B—N5—C5A—C6	−179.95 (3)	C4B'—N5'—C5A'—C6'	179.31 (3)
C4B—N5—C5A—C9A	−0.51 (3)	C4B'—N5'—C5A'—C9A'	−0.35 (3)
C4B—C10A—N10—C9A	−1.55 (3)	C4B'—C10B—N10'—C9A'	−1.60 (3)
C4B—C10A—C11—C11A	−1.89 (2)	C4B'—C10B—C11'—C11B	1.01 (2)
N5—C4B—C4A—C11A	177.73 (3)	N5'—C4B'—C4A'—C11B	177.78 (3)
N5—C4B—C10A—N10	2.37 (3)	N5'—C4B'—C10B—N10'	0.20 (3)
N5—C4B—C10A—C11	−176.68 (3)	N5'—C4B'—C10B—C11'	−178.78 (3)
N5—C5A—C6—C7	178.59 (3)	N5'—C5A'—C6'—C7'	−178.58 (3)
N5—C5A—C9A—C9	−179.01 (3)	N5'—C5A'—C9A'—C9'	178.00 (3)
N5—C5A—C9A—N10	1.20 (3)	N5'—C5A'—C9A'—N10'	−1.02 (3)
C5A—N5—C4B—C10A	−1.27 (3)	C5A'—N5'—C4B'—C10B	0.76 (3)
C5A—C6—C7—C8	0.46 (3)	C5A'—C6'—C7'—C8'	0.00 (4)
C5A—C9A—C9—C8	0.41 (3)	C5A'—C9A'—C9'—C8'	1.15 (3)
C5A—C9A—N10—C10A	−0.04 (3)	C5A'—C9A'—N10'—C10B	2.00 (3)
C6—C5A—C9A—C9	0.43 (3)	C6'—C5A'—C9A'—C9'	−1.67 (3)
C6—C5A—C9A—N10	−179.36 (3)	C6'—C5A'—C9A'—N10'	179.32 (3)
C6—C7—C8—C9	0.40 (4)	C6'—C7'—C8'—C9'	−0.55 (4)
C7—C6—C5A—C9A	−0.87 (3)	C7'—C6'—C5A'—C9A'	1.09 (4)
C7—C8—C9—C9A	−0.84 (3)	C7'—C8'—C9'—C9A'	−0.03 (4)
C8—C9—C9A—N10	−179.80 (3)	C8'—C9'—C9A'—N10'	−179.84 (3)
C9—C9A—N10—C10A	−179.83 (3)	C9'—C9A'—N10'—C10B	−177.01 (3)
C9A—N10—C10A—C11	177.22 (2)	C9A'—N10'—C10B—C11'	177.10 (2)
N10—C10A—C11—C11A	179.27 (3)	N10'—C10B—C11'—C11B	−177.76 (3)
C10A—C4B—C4A—C11A	−0.15 (2)	C10B—C4B'—C4A'—C11B	−0.69 (2)
C11—P1—O2—C12	177.92 (2)	C11'—P1'—O2'—C12'	65.76 (2)
C11—P1—O3—C13	−67.20 (2)	C11'—P1'—O3'—C13'	57.67 (2)

### (4b) Hydrogen-bond geometry (Å, º)

**Table:**

*D*—H···*A*	*D*—H	H···*A*	*D*···*A*	*D*—H···*A*
C9—H9···O1′	1.077 (5)	2.341 (5)	3.1776 (4)	133.3 (4)
N10—H10···O1	1.019 (5)	2.343 (6)	2.9896 (3)	120.3 (4)
N10—H10···O1′	1.019 (5)	1.911 (6)	2.8468 (3)	151.2 (5)
C9′—H9′···O1	1.077 (5)	2.540 (5)	3.2887 (4)	125.8 (4)
N10′—H10′···O1	1.015 (6)	1.956 (6)	2.8741 (3)	148.9 (5)
N10′—H10′···O1′	1.015 (6)	2.290 (6)	2.9340 (3)	120.1 (4)
C4—H4···O3′^i^	1.079 (5)	2.398 (5)	3.2761 (4)	137.5 (4)
C8′—H8′···O2^ii^	1.079 (5)	2.416 (5)	3.3475 (4)	143.8 (4)
C13′—H13*e*···N5′^iii^	1.075 (6)	2.588 (6)	3.6340 (5)	164.1 (5)

Symmetry codes: (i) −*x*, −*y*+1, −*z*+1; (ii) −*x*+1, −*y*+1, −*z*+1; (iii) −*x*+1, −*y*+1, −*z*+2.
